# Photoactive Hydrogels Containing Merocyanine/Spiropyran Units for Wound Dressing Applications

**DOI:** 10.1021/acs.biomac.5c01956

**Published:** 2025-12-29

**Authors:** Hitesh Katariya, Ewa Chrzescijanska, Krzysztof Jerczynski, Jan Rutkowski, Vishal Purohit, Magdalena Lipinska, Ahmet Cetinkaya, Ann-Kathrin Kissmann, Daniel Gruber, Jan-Christoph Walter, Frank Rosenau, Aleksandra Maciejczyk, Agata Przekora-Kuśmierz, Jozef Kollár, Jaroslav Mosnáček, Joanna Pietrasik

**Affiliations:** † Institute of Polymer and Dye Technology, 49584 Lodz University of Technology, Stefanowskiego 16, Lodz 90-537, Poland; ‡ Faculty of Chemistry, Institute of General and Ecological Chemistry, Lodz University of Technology, Zeromskiego 116, Lodz 90-924, Poland; § Department of Chemical Sciences, P. D. Patel Institute of Applied Sciences, Charotar University of Science and Technology (CHARUSAT), Changa, 388 421 Gujarat, India; ∥ Institute of Pharmaceutical Biotechnology, 98917 Ulm University, Meyerhofstrasse 1, Ulm 89081, Germany; ⊥ Department of Tissue Engineering and Regenerative Medicine, 49554 Medical University of Lublin, Chodzki 1, Lublin 20-093, Poland; # Polymer Institute of the Slovak Academy of Sciences, Dubravska Cesta 9, Bratislava 845 41, Slovakia

## Abstract

Chronic wounds present alkaline, microbially burdened environments that impede tissue repair and necessitate materials capable of actively modulating local chemistry. Here, we report the synthesis and characterization of light-responsive hydrogels incorporating a methacrylate-functionalized merocyanine photoacid (E-MCMAH^+^) within the OEGMA/EGDMA networks to enable spatiotemporal control over acidity. Systematic variation of photoacid loading revealed its critical role in governing network swelling, mechanical integrity, and optical transitions associated with spiropyran–merocyanine interconversion. Solid-state UV–vis spectroscopy coupled with Tauc analysis elucidated distinct band gap features arising from photoisomerization. Under visible and UV irradiation, the hydrogels exhibited robust, reversible proton release, which translated to enhanced ion mobility confirmed electrochemically. Cytocompatibility studies demonstrated high fibroblast viability in the dark and only transient photochemical effects under blue light. Notably, the materials displayed inherent antibacterial activity against *Pseudomonas aeruginosa* and MRSA, which intensified to complete eradication under illumination. These properties position E-MCMAH^+^ hydrogels as versatile platforms for advanced wound dressings.

## Graphical abstract

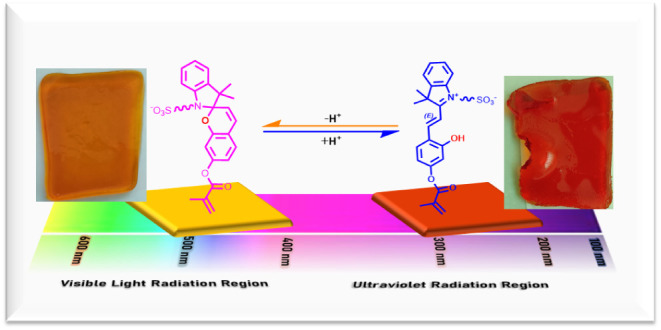

## 1 Introduction

Chronic wounds, particularly those associated with diabetes or vascular insufficiencies, often present with elevated alkaline pH levels, typically ranging from 7.5 to 8.9,^1,2^ along with excessive exudate and microbial colonization. Together, these factors delay tissue repair and increase the risk of complications.^1–3^ Among various intelligent materials, stimuli-responsive hydrogels offer exceptional promise due to their high water content, softness, and biocompatibility-mimicking the native extracellular matrix while allowing for the incorporation of dynamic functionalities.^4–7^ Among external stimuli, light has emerged as an ideal trigger for biomedical systems, enabling precise, remote, and noninvasive control over hydrogel behavior.^4–7^ Recent developments in photoresponsive hydrogels have largely centered around the use of molecular photoswitches such as azobenzenes,^8–10^ coumarins,^11,12^ and spiropyrans.^13,14^

Spiropyrans (SP) are especially attractive due to their reversible isomerization between a closed, colorless spiropyran (SP) form and a highly conjugated, colored merocyanine (MC) form induced by UV light/visible light irradiation.^13–16^ This transformation is accompanied by pronounced changes in polarity, dipole moment, and acidity, making SP-based systems capable of modulating not only optical but also chemical and physical properties of materials.^16,17^ The reversible photoisomerization between the open-ring merocyanine form (MCMA) and closed-ring spiropyran form (SPMA), encompassing also protonated E and Z isomers, is shown in Figure 1. In this process, UV irradiation facilitates ring opening accompanied by proton uptake, resulting in elevated pH, whereas visible light irradiation induces ring closure and proton liberation, thereby diminishing pH and modulating molecular polarity, dipole moment, and acidity.^18^ This photochemical mechanism aligns with the isomerization kinetics observed in multiresponsive hydrogels, wherein copolymer composition (e.g., incorporation of acrylic acid to foster acidic microenvironments) and the light-induced disaggregation of H-aggregated merocyanine under 405 nm excitation account for the triexponential fluorescence decay profiles, thereby affording precise regulation of optical, chemical, and physical attributes in materials tailored for photoactuators and nonlinear optical applications.^18^ Beyond traditional optical switching, the ability of spiropyrans to act as reversible photoacids releasing and reclaiming protons under light exposure has recently garnered attention for applications requiring local pH modulation.^19^ This reversible, metastable-state photoacidity is particularly appealing in biological systems where temporal control over acidity could influence cell behavior, drug release, or microbial suppression.^19^ Unlike excited-state photoacids with short lifetimes, metastable-state photoacids (mPAHs) maintain their proton-donating state for seconds to minutes, allowing more sustained and controllable pH changes under biologically relevant conditions.^20^ In addition to their chemical responsiveness, certain spiropyran derivatives have been reported to exhibit bioactivity, such as antibacterial properties, further enhancing their utility in wound dressings.^17^ The design and substitution of electron-donating and electron-withdrawing groups on the SP scaffold have been shown to finely tune switching kinetics, photostability, and acidity, offering a toolbox for custom-tailored behavior in responsive materials.^15–17,19,21^

**Figure 1:**
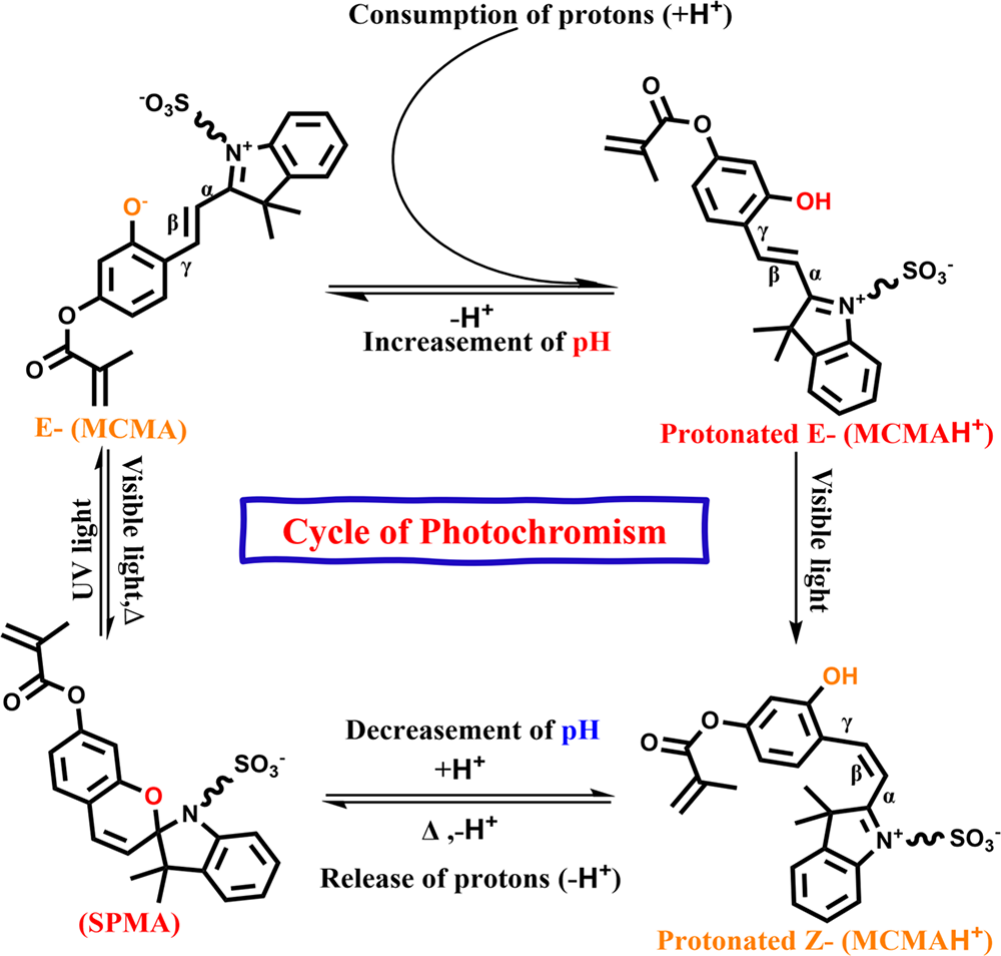
Cycle of photochromism of merocyanine (MCMA)/spiropyran (SPMA) forms.

Despite these advances, most current SP-based systems emphasize optical or mechanical responses, and relatively few works fully exploit the reversible proton release characteristic of mPAHs within hydrogel matrices. Moreover, challenges such as poor aqueous solubility, photoinstability, or weak polymer integration often limit their practical deployment. Previous studies have demonstrated that spiropyran-based systems can exhibit strong photoactivation, minimal photofatigue, and biocompatibility-properties considered essential for wound healing applications.^21^ Those systems utilized a covalent anchoring strategy to prevent dye leaching and enhance long-term operational stability. Moreover, other research has shown that spiropyran-based electrochemical composites can be engineered to support dual functionalities, such as light-triggered proton release and bioelectrical signal transduction, thereby expanding their utility in smart wound care platforms.^22–25^

Recently, stimulus-responsive hydrogels capable of modulating the wound microenvironment have emerged as promising antibacterial materials. Light-, thermal-, and pH-responsive systems can suppress bacterial proliferation by altering local conditions or generating reactive species while minimizing the risk of bacterial resistance.^26,27^ In particular, researchers highlighted that environmental stimuli, such as light and pH, play a crucial role in developing next-generation antibacterial wound dressings.^28^ In this context, spiropyran-based photoacid systems offer a unique mechanism, wherein the light-induced conversion from the merocyanine to spiropyran form releases protons and transiently lowers the surrounding pH. Such localized pH modulation can impair bacterial cell membranes and metabolic processes, thus imparting a controllable antibacterial effect without the need for conventional antibiotics. This can make spiropyran-functionalized hydrogels highly attractive for multifunctional wound dressings, combining photoresponsivity and inherent antimicrobial potential.

Herein, we report the design, synthesis, and properties of a hydrogel containing a novel merocyanine-based methacrylate photoacid, (E)-3-(2-(2-hydroxy-4-(methacryloyloxy)­styryl)-3,3-dimethyl-3H-indol-1-ium-1-yl)­propane-1-sulfonate (E-MCMAH^+^). This monomer was copolymerized with oligo­(ethylene glycol) methyl ether methacrylate (OEGMA) and ethylene glycol dimethacrylate (EGDMA) and formed a covalent hydrogel network, in which a sulfonate group within the photoacid provided enhanced aqueous compatibility, while the merocyanine and spiropyran functionalities were associated with photoacid activities. A varied concentration of photoacid was introduced into the gel structure to vary its properties. Swelling tests and viscoelastic properties were used for the validation of the physical performance of the gels. while the photoactivity was investigated by using solid-state UV spectroscopy and Tauc’s equation for the energy band gap. Thus, cyclic voltammetry and differential potentiometry pulse were carried out in the dark and under a light source to confirm the ionic transport and ionic conductivity. Biological evaluations substantiated the hydrogel’s suitability for wound-dressing applications. The material was noncytotoxic in ISO 10993-5 assays and confocal imaging, exhibited acceptable phototoxicity under blue-light irradiation, and supported normal fibroblast morphology. It further demonstrated inherent antibacterial activity against *P. aeruginosa* and MRSA, which intensified to complete eradication upon light activation via synergistic proton release and ROS generation. Collectively, these results confirm the hydrogel’s potential as a multifunctional wound-care material.

## 2 Experimental Section

### 2.1 Materials

2,4-Dihydroxybenzaldehyde (Thermo Fisher, 98%), triethylamine (Sigma-Aldrich, ≥99%), 1,3-propane sultone (Sigma-Aldrich, 98%), methacryloyl chloride (Sigma-Aldrich, 97%), ethyl alcohol (POCH, 98%), dichloromethane (PureLand, 99.7%), ethyl acetate (POCH, 99.8%), *n*-hexane (Chempur, 99%), anhydrous dimethyl sulfoxide (DMSO, Sigma-Aldrich, 99.9%), magnesium sulfate (MgSO_4_, Sigma-Aldrich, 99.8%), 2,2′-azobis­(2-methylpropionitrile) (AIBN, Sigma-Aldrich, 98%), and 2-cyano-2-propyl benzodithioate (CPDB, Sigma-Aldrich, >97%) were used as received without further purification.

Poly­(ethylene glycol) methyl ether methacrylate (OEGMA, average *M*_n_ ≈ 300, stabilized with ∼300 ppm BHT) and ethylene glycol dimethacrylate (EGDMA, 98%, containing 90–110 ppm monomethyl ether hydroquinone) were passed through a column of basic activated alumina prior to use to remove inhibitors.

### 2.2 Synthesis of (E)-3-(2-(2-hydroxy-4-(methacryloyloxy)­styryl)-3,3-dimethyl-3H-indol-1-ium-1-yl)­propane-1-sulfonate (E-MCMAH^+^)

A three-step procedure was applied (Figure S1).^29^ 4-Formyl-3-hydroxyphenyl methacrylate and 3-(2,3,3-trimethyl-3H-indol-1-ium-1-yl)­propane-1-sulfonate were mixed in ethanol at a molar ratio of 1.5:1 and stirred for 6 h at 90 °C under an inert atmosphere. After the mixture cooled, the precipitates were filtered and washed with cold ethanol several times under high vacuum filtration. The E-MCMAH^+^ was collected as a tangy orange solid in 72% yield [^1^H NMR (400 MHz, DMSO-d_6_) (Figure S2) δ 11.40 (s, 1H), 8.51 (d, 1H), 8.33 (d, 1H), 8.04–7.94 (m, 1H), 7.88–7.72 (m, 2H), 7.71–7.52 (m, 2H), 6.85–6.72 (m, 2H), 6.30–6.22 (m, 1H), 5.90 (dp, 1H), 4.77 (t, 2H), 2.60 (dt, 2H), 2.20 – 2.10 (m, 2H), 2.01–1.92 (m, 3H), 1.73 (s, 6H)].

### 2.3 Synthesis of oly­(oligo­(ethylene glycol) methyl ether methacrylate)-co-poly­((e)-3-(2-(2-hydroxy-4-(methacryloyloxy)­styryl)-3,3-dimethyl-3H-indol-1-ium-1-yl)­propane-1-sulfonate)-co-ethylene glycol dimethacrylate, by Free Radical Polymerization

The merocyanine-containing gel was synthesized via free radical polymerization using OEGMA (1.022 g, 3.4075 mmol) and merocyanine-based methacrylate photoacid (400.00 mg, 0.8519 mmol) as monomers, EGDMA (16.89 mg, 0.0852 mmol) as a cross-linker, and AIBN (4.66 mg, 0.0284 mmol) as a thermal initiator. All components were dissolved in DMSO to obtain a homogeneous solution, which was subsequently degassed under an argon atmosphere for 20 min. The degassed solution was transferred into a 1 mL syringe and injected into a mold prepared by sandwiching a 1 mm-thick spacer between two clean glass plates. The mold edges were sealed by using a silica-based sealant to prevent leakage and solvent evaporation. The assembly was placed in a preheated oven at 70 °C for 12 h to allow for complete gelation. After cooling to room temperature, the hydrogel sheet was carefully removed from the glass plates and sequentially washed to remove unreacted components: it was washed twice with diethyl ether to ensure the elimination of residual OEGMA, followed by four washes with cold ethanol to extract unreacted E-MCMAH^+^. The ethanol washings were collected and evaporated to dryness under reduced pressure, and the residual E-MCMAH^+^ was quantified using a high-precision analytical balance. The extent of photoacid incorporation was determined by subtracting the mass of unreacted E-MCMAH^+^ from the initial monomer feed, and the incorporation efficiency was expressed as a weight percentage relative to the total dry mass of the hydrogel. Finally, the purified gel was dried overnight in a high-vacuum oven to yield the final dried gel for further characterization.

### 2.4 Characterization Techniques

#### 2.4.1 Proton Nuclear Magnetic Resonance

Proton nuclear magnetic resonance (^1^H NMR) spectroscopy was used to determine the structure of the obtained monomer. The ^1^H NMR spectra were recorded on a Bruker Avance DPX 700 MHz. Samples were prepared by dissolving the analyzed samples in DMSO-d6.

#### 2.4.2 Swelling Tests

The swelling degree of the obtained gels was calculated by immersing the weighed amounts of the gels in PBS buffer (pH 7.40) for 48 h at room temperature. The mass of the sample was determined before and after swelling, and next, the swelling degree was calculated based on the equation:

$$\mathrm{Swelling degree}\;\%=\frac{W_{s}-W_{d}}{W_{d}}\times 100$$

Here, *W*_s_ represents the weight of the swollen gel, and *W*_d_ represents the weight of the dry gel.

#### 2.4.3 Dynamic Rheological Measurements

Dynamic measurements of viscoelastic properties were carried out under shear using an Ares G2 oscillation rotational rheometer, TA Instruments (USA), with two parallel plates (*d* = 25 mm). Prior to the measurements, films were dried in a vacuum oven at 50 °C and 10 mbar for 2 h. Sample thickness was determined according to data from the rheometer Ares G2 software, and it was 1.5 ± 0.22 mm. The amplitude sweep tests at a constant applied frequency range of 10 rad/s and oscillation strain up to 5% and 10% were performed. The average values of storage shear modulus *G*’ (Pa), loss shear modulus *G*” (Pa), tan δ (−), complex viscosity η’ LVR (Pa·s), and dynamic viscosity η’ (Pa·s) for the linear viscoelastic range up to 5% of oscillation strain were calculated. The frequency sweep tests were performed for a constant value of 0.1% oscillation strain to determine the viscoelastic parameters as a function of angular frequency in the range of 0.1–628 rad/s. The measurements were performed at ambient temperature.

#### 2.4.4 Solid-State UV–Vis Spectroscopy

Solid-state UV–vis spectroscopy was employed to monitor the reversible photoisomerization behavior of the photoacid-containing hydrogel. Initially, a hydrogel composed of P­(OEGMA-co-E-MCMAH^+^) was synthesized and stored in the dark to maintain the photoacid in its merocyanine form. A control gel containing only P­(OEGMA) was also prepared under identical conditions. The dried samples were analyzed in the solid state using UV–vis spectroscopy over the 200–1000 nm range to establish baseline absorbance profiles. To induce conversion to the spiropyran form, the merocyanine-containing gel was immersed in anhydrous DMSO to facilitate swelling, followed by irradiation under a blue light source (λ = 460 nm) using a lamp with an intensity of 14 mW/cm^2^. The sample was continuously irradiated for 5 h, after which it was dried under reduced pressure (10 mbar) at 35 °C to remove solvent and fix the spiropyran form in the solid state. UV–vis spectra were recorded again to capture the optical changes associated with the ring-closed spiropyran structure. For reconversion back to the merocyanine state, the spiropyran-containing gel was immersed in 0.1 M aqueous HCl to promote protonation, followed by UV light exposure (λ = 365 nm) to induce ring-opening. The gel was then dried at 35 °C under a 10 mbar pressure. The resulting dried gel sample, now in the photoreverted merocyanine form, was once again analyzed by solid-state UV–vis spectroscopy to confirm the reversibility of the E-MCMAH^+^ ↔ SPMA transition and the retention of photoacid function.

#### 2.4.5 Electrochemical Measurements

Methods of cyclic voltammetry (CV) and differential pulse voltammetry (DPV) were used with an Autolab PGSTAT30 Electrochemical Analyzer (EcoChemie, Netherlands).

In water solutions, a three-electrode cell system, including a saturated calomel electrode (SCE) as a reference electrode, a platinum wire as an auxiliary electrode, and the platinum (geometric surface area of 0.5 cm^2^) as the working electrode, was applied in the electrochemical studies. Electrooxidation and electroreduction of compounds were performed by CV and DPV within the potential range from 0 to 1.2 V, from 0 to −1.0 V, or from 0 to −0.9 V, respectively.

In anhydrous solutions (DMSO), a three-electrode system consisted of a reference electrode, an auxiliary platinum wire electrode, and a platinum strip working electrode with a geometric surface area of 0.5 cm^2^. A potential of the working electrode was measured vs ferricinium/ferrocene reference electrode (Fc^+^/Fc) couple, as recommended by the IUPAC.^68,69^ The reference electrode was a platinum wire immersed in a solution of ferrocene (*c* = 1 × 10^–3^ mol/L in 0.1 mol L^–1^ (C_4_H_9_)_4_NClO_4_ in DMSO) and placed in a glass tube with a tiny hole (diameter ≈ 0.2 mm) at the bottom. This hole ensured electrochemical contact between the electrolyte in the reference electrode compartment and the electrolyte in the reactor compartment. The next step was coulometric oxidation to obtain an equivalent concentration of ferrocinium ion (Fc^+^) (c_Fc_ = c_Fc+_). The electrode prepared in this manner was used in our studies as a reference electrode.

Electrooxidation and electroreduction of compounds were performed by CV and DPV within the potential range from 0 to 2.0 V and from 0 to −1.8 V.

All CV curves were recorded with scan rates ranging from 0.1 V/s. The modulation amplitude of 25 mV and a pulse width of 50 ms were applied for DPV measurements. All experiments were carried out at room temperature. Before measurements, the solutions were purged with argon to remove dissolved oxygen. During measurements, an argon blanket was kept over the solutions.

#### 2.4.6 *In Vitro* Cytotoxicity Tests

An indirect cytotoxicity test was performed in accordance with the procedure described in ISO 10993-5. Human skin fibroblasts (BJ cell line purchased from ATCC) were seeded into 96-well plates in 100 μL of culture medium at a density of 3 × 10^4^ cells per well and cultured for 24 h. Then, the culture medium was replaced with extracts of samples prepared according to ISO 10993-12. Biomaterial extracts were prepared by immersing biomaterials in a complete culture medium (keeping a ratio of 50 mg of the gel per 1 mL of the medium), followed by their incubation at 37 °C for 24 h. The culture medium incubated in the polystyrene well of the 24-well plate was treated as a negative control for cytotoxicity (sample marked as control). The BJ cells were exposed to the extracts for 24 h, and then the MTT (Sigma-Aldrich, Poland) assay was performed to assess cell viability. The MTT assay is a colorimetric assay for assessing cell metabolic activity. The obtained results were expressed as a percentage of the absorbance value of the negative control for cytotoxicity.

To evaluate the cytotoxicity of the gel in direct contact with the cells, the BJ fibroblasts were seeded into a 24-well plate in 500 μL of the culture medium at a density of 2 × 10^5^ cells/well and cultured for 24 h. Then, the tested sample (25 mg) was gently placed on the monolayer of the cells, and the plate was returned to the cell culture incubator for 48 h. After incubation, cells were stained using the Live/Dead Double Staining Kit (Sigma-Aldrich Chemicals, Poland) following the manufacturer’s instructions. This kit contains calcein-AM, which labels viable cells with green fluorescence, and propidium iodide, which marks dead cells with red fluorescence. Cell viability was subsequently analyzed and visualized by confocal laser scanning microscopy (CLSM).

#### 2.4.7 *In Vitro* Phototoxicity Test

The test was performed to assess how gel irradiation affects cell viability. For this purpose, the BJ cells were seeded into Petri dishes (with a diameter of 3.5 cm) in 3 mL of the culture medium at a density of 8 × 10^5^ cells per well and cultured for 24 h. Then, the tested sample was gently placed on the monolayer of the cells, with a ratio of 50 mg of the sample per 1 mL of culture medium. The Petri dishes were irradiated with blue light (460 nm) for 2 and 3 h. The dishes kept in the dark served as controls. After exposure to blue light, cell viability was determined using a Cell Counting Kit-8 (CCK-8, Sigma-Aldrich Chemicals), which utilizes the water-soluble tetrazolium salt. Additionally, after 3 h of exposure to blue light, the BJ cells cultured in the presence of the gel were left in the incubator in the dark for a further 12 h to determine whether cell viability would decrease over time.

#### 2.4.8 Antimicrobial Test

*P. aeruginosa* PAO1 was cultivated in 5 mL of LB medium (Carl Roth GmbH + Co. KG, Karlsruhe, Germany), and *S. aureus* MRSA was cultivated in 5 mL of Todd-Hewitt medium at 37 °C for 16 h while shaking at 150 rpm. For antibacterial testing, the experiments were conducted either in the dark or under blue light irradiation (350 mA). Equal-sized gel pieces (2 mm diameter) were prepared and equilibrated for use in the aqueous phase by washing twice with 200 μL of 70% ethanol and three times with 200 μL of sterile Milli-Q water.^30^ Both hydrogels and bare polystyrene cell culture material (negative control) were inoculated with 1.5 × 10^5^ *P. aeruginosa* PAO1 cells and incubated for 24 h at 37 °C under gentle shaking. After incubation, planktonic phases were transferred to fresh 96-well plates, and the optical density at 600 nm was measured using a Tecan Infinite F200 microplate reader (Tecan Group Ltd., Männedorf, Switzerland). Cell viability was further assessed via a resazurin reduction assay,^31^ 20 μL of sterile resazurin solution (0.15 mg/mL) was added to the cultures, incubated for 2 h at 37 °C, and the fluorescence of the resulting resorufin was measured at 535 nm excitation and 595 nm emission using a Tecan Infinite F200 microplate reader (Tecan Group Ltd., Männedorf, Switzerland).

#### 2.4.9 Photosensitizing Capability

Reactive oxygen species (ROS) generation was quantified using the Amplex Red Hydrogen Peroxide/Peroxidase Assay Kit (Thermo Fisher Scientific, Waltham, USA). Reagent preparation and concentrations (Amplex Red, HRP, Reaction Buffer, and H_2_O_2_ standards) were performed according to the manufacturer’s protocol. For each experiment, 50 μL of PBS containing either a gel sample, 10 μM H_2_O_2_ (positive control), or PBS alone (blank) was incubated for 2 h at 37 °C. This procedure was carried out twice, once under blue-light exposure and once in complete darkness. After incubation, 50 μL of freshly prepared 100 μM Amplex Red and 0.2 U/mL horseradish peroxidase working solution were added to each well. After 30 min of incubation, protected from light, the fluorescence was recorded on a Tecan Infinite F200 microplate reader (Tecan, Männedorf, Switzerland) using 535 nm excitation and 595 nm emission. Background signals were corrected by subtracting the blank values, and all measurements were performed in triplicate.

#### 2.4.10 pH Modulation Procedure

For the pH modulation study, a merocyanine-functionalized hydrogel, Gel-1, was used, differing only in the content of the covalently embedded photoacid. The sample was prepared according to the following procedure: 181.6 mg of Gel-1 was transferred into a clean 30 mL borosilicate glass vial. The hydrogel was immersed in 20.0 mL of ultrapure Milli-Q water (resistivity ≥ 18.2 MΩ·cm) and allowed to equilibrate under ambient laboratory conditions (22 ± 1 °C) in the dark for 30 min prior to light exposure. The photoirradiation was performed using an array of blue LED diodes (λ_max_ = 460 nm), connected to a regulated DC power supply (UNI-T, UTP3315TFL-II) delivering a constant output of 3.18 V and 0.440 A. The light intensity was maintained at 14 mW/cm^2^, and the distance between the light source and the sample surface was fixed at 3 cm. Irradiation was carried out for 60 min under static conditions. The pH of the aqueous phase above the gel surface was monitored at 10-min intervals using a calibrated benchtop pH meter equipped with a microelectrode. The tip of the electrode was positioned directly above the gel surface and remained immersed in the surrounding solution throughout the measurement, avoiding any physical contact or stirring of the gel. No additional agitation or sample handling was performed during or after the irradiation process. All measurements were conducted in triplicate to ensure reproducibility.

## 3 Results and Discussion

### 3.1 Molecular Characterization of the Gels

The synthetic route for developing multifunctional hydrogels is illustrated in Scheme 1. The hydrogels were synthesized by free radical copolymerization (FRP) of oligo­(ethylene glycol) methyl ether methacrylate (OEGMA) and (E)-3-(2-(2-hydroxy-4-(methacryloyloxy)­styryl)-3,3-dimethyl-3H-indol-1-ium-1-yl)­propane-1-sulfonate (E-MCMAH^+^) as monomers, and ethylene glycol dimethacrylate (EGDMA) as a cross-linker. E-MCMAH^+^ was synthesized according to the procedure described previously (Figure S1).^29^ The ^1^H NMR spectrum confirming the chemical structure of (E-MCMAH^+^) (Figure S2) shows characteristic signals at δ 6.85–6.72 (m, 2H), corresponding to the conjugated double bond system formed in the merocyanine core during synthesis.

**Scheme 1:**
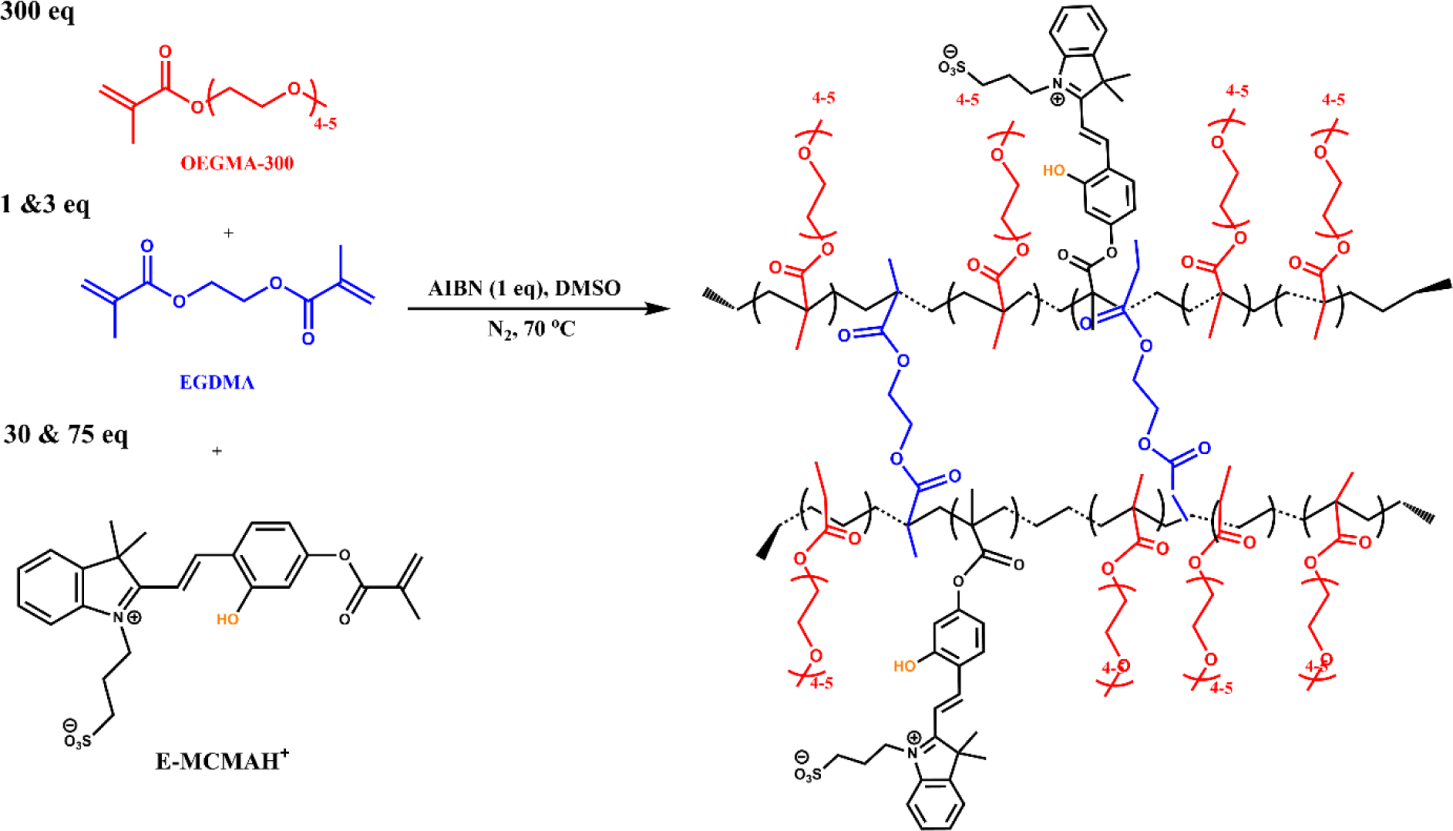
Synthetic Procedure for the P­(OEGMA-co-E-MCMAH^+^) Gel Formation

The initial monomer feed compositions were varied to introduce different concentrations of photoacid within the gel structure. Specifically, four formulations were designed: Gel-0, composed only of OEGMA units cross-linked with EGDMA; Gel-1, with 10 mol % photoacid vs OEGMA; Gel-2, with 25 mol % photoacid; and Gel-3, with 10 mol % photoacid but with a higher cross-linker concentration (Table 1). Higher photoacid unit content was expected to enhance the light-responsive proton release capacity of the gel, facilitating localized pH modulation. The experimentally determined photoacid content in the hydrogels was lower than the corresponding feed ratios (Table 1). This discrepancy is attributed primarily to the reduced copolymerization reactivity of the merocyanine-based methacrylate (E-MCMAH^+^) compared to that of the OEGMA. Steric hindrance introduced by the bulky chromophore around the methacrylate double bond further decreases its incorporation efficiency. Although the merocyanine moiety is hydrophilic, its delocalized charge and electronic structure can limit diffusion and accessibility to the propagating radicals within the OEGMA-EGDMA network, resulting in incomplete incorporation. In addition, partial removal of unreacted or physically bound photoacid during the postcuring washing step contributes to the observed deviation. These factors collectively explain the lower experimentally determined photoacid content relative to the theoretical feed, in agreement with previous reports for chromophore-functionalized methacrylate monomers.^32,33^ Concurrently, the hydrophilic OEGMA units were expected to ensure adequate swelling and water retention capability, while chemical cross-linking with EGDMA maintained mechanical integrity, preventing excessive swelling that could compromise gel stability or adhesion to wound sites. The formation of the gels, either based on pure OEGMA (Figure 2A) or E-MCMAH^+^/OEGMA mixtures (Figure 2B), was carried out by casting the monomer mixture between glass plates and curing overnight under an inert argon atmosphere to ensure controlled network formation and prevent oxygen inhibition. The resulting hydrogels formed as uniform, thin films with a consistent thickness of approximately 1 mm.

**Table 1:** Molar Feed Ratios of Monomers, Cross-Linker, and Initiator Used for the Preparation of Reference Hydrogel (Gel-0) and Photoresponsive Hydrogels (Gel-1, Gel-2, and Gel-3)

	OEGMA	E-MCMAH^+^	EGDMA	AIBN
Gel-0*	300	-	1	1
Gel-1	300	30	1	1
Gel-2	300	75	1	1
Gel-3	300	30	3	1

**Figure 2:**
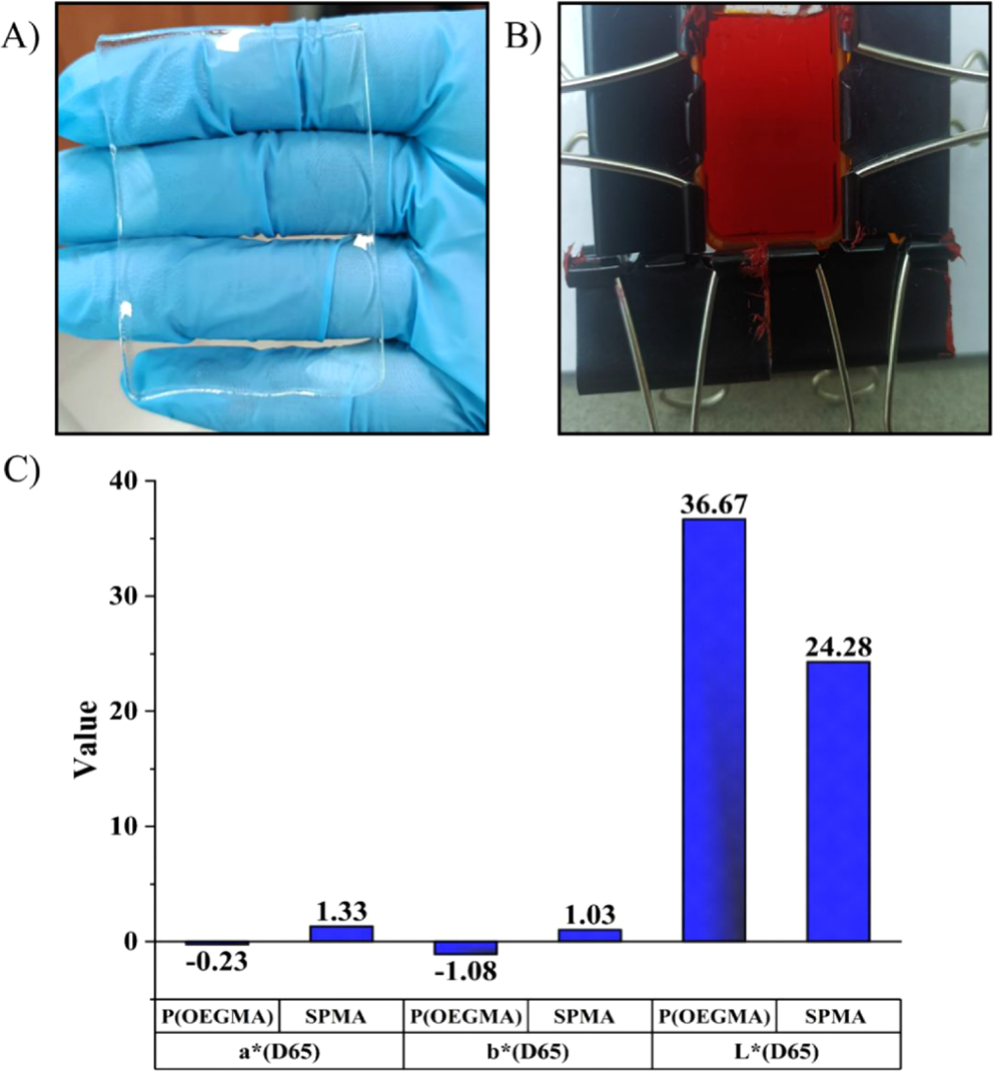
(A) Photograph of the transparent POEGMA gel. (B) Setup for the synthesis of P­(OEGMA-co-E-MCMAH^+^) gel. (C) Spectrophotometric analysis of Gel-1 and reference Gel-0.

The morphological integrity of the gels was verified through digital microscopy analysis, which revealed the formation of homogeneous networks without visible phase separation or defects (Figure S3). The incorporation of the photoacid monomer containing merocyanine groups into the gel structure was visually apparent through distinct color changes observed by the naked eye (Figure S3B). Moreover, Figure 2C shows spectrophotometric data obtained for the reference and photoacid-containing gels. The orangish color, confirmed by *a, b,* and *L* parameters, certified that E-MCMAH^+^ was successfully incorporated into the network structure. The amount of photoacid incorporated into the hydrogel was estimated gravimetrically (see SI). For Gel-1, this analysis revealed an incorporation of approximately 6.25 mol % (5.87 wt %) of the photoacid monomer within the polymer network. Increasing the photoacid feed to 25 mol % (Gel-2) resulted in 13.6 mol % (12.8 wt %) embedded photoacid monomer in the final gel. Gel-3, having the same 10 mol % photoacid feed as Gel-1 but a different cross-linker concentration, exhibited similar incorporation of E-MCMAH^+^, 6.6 mol % (6.25 wt %), as compared to Gel-1. Taken together, the synthesis of structurally robust, multifunctional hydrogels with determined photoacid content was confirmed.

### 3.2 Swelling Behavior and Light Responsiveness of the Gels

Figure 1 highlights the photochemical cyclic interconversion between the SPMA and protonated E-MCMAH^+^ forms, driven by visible light. The merocyanine form introduces ionizable groups and facilitates local proton release, increasing the internal electrostatic repulsion and osmotic pressure within the hydrogel network. It further promotes additional water uptake and network expansion. Therefore, the swelling behavior of the merocyanine-based hydrogels was investigated at physiological pH (phosphate buffer, pH 7) under two distinct conditions: in the absence of light (dark) and under blue light irradiation (λ = 460 nm, intensity = 14 mW/cm^2^). The swelling ratio %, defined as the weight of the swollen gel divided by the weight of the dry gel, was used to assess water uptake and the gel’s responsiveness to light.

It appeared that Gel-1 exhibited highly pronounced light-responsive swelling behavior. In the dark, the swelling ratio reached 1040%, which further increased to 1420% under light irradiation (Figure 3A). Gel-1 thickness in the dry state was measured by using digital microscopy to be approximately 0.960 ± 0.012 mm (Figure 3B). After irradiation and subsequent swelling, the gel thickness increased significantly to 2.280 ± 0.046 mm, corresponding to more than a 2-fold increase in its physical dimension (Figure 3C). This change in thickness was consistent with the gravimetrically determined swelling ratio and supports the concept of phototriggered volumetric expansion. In contrast, Gel-2, with a higher photoacid content (13.6 mol %), showed a more limited swelling capacity, reaching 560% in the dark and 770% in the presence of light (Figure 3A). In this case, the reduced swelling behavior might be attributed to the increased hydrophobicity of that material, although overall changes in the swelling were similar to those of Gel-1 (1.4 times). Gel-3 (MCMAH^+^ 6.6 mol %) exhibited swelling ratios of 840% in the dark and 990% under light irradiation (Figure 3A). The relatively small difference between dark and light conditions suggests that the increased cross-linking density restricted the flexibility of the network and limited the conformational changes needed for efficient spiropyran activation and subsequent swelling enhancement. Therefore, this sample was excluded from further studies. It was concluded that a composition of both Gel-1 and Gel-2 provides a reasonable swelling increase under light due to efficient spiropyran activation and sufficient network mobility.

**Figure 3:**
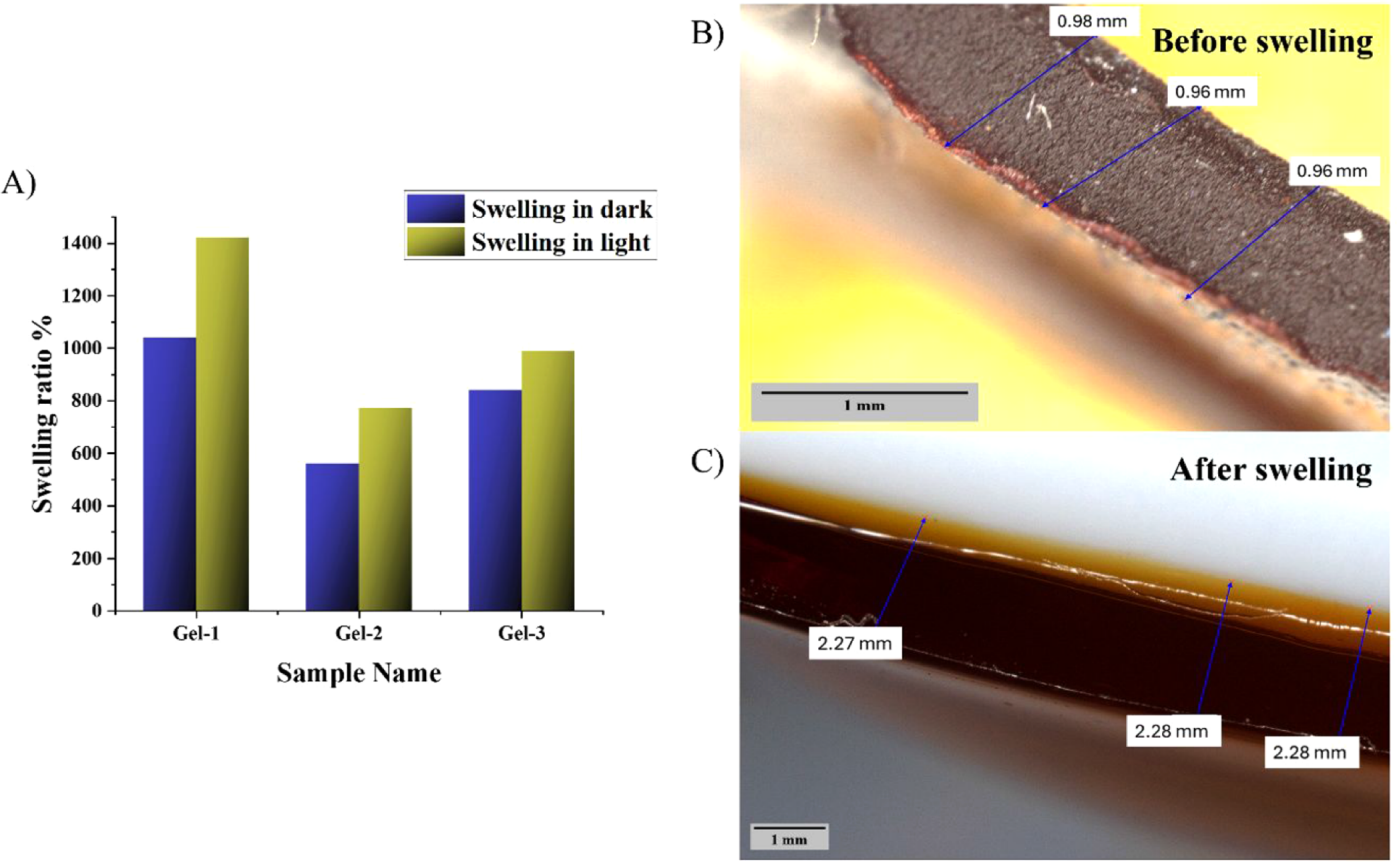
(A) Swelling ratios in the dark and light for the investigated gels. (B) Digital microscopy image of unswollen Gel-1. (C) Digital microscopy image of swollen Gel-1.

### 3.3 Rheological Characterization of the Gels

The viscoelastic properties of the investigated Gel-1 and Gel-2 were assessed through oscillatory rheology in both dry and swollen states (both in the E-MCMAH^+^ form) to understand how photoacid influences mechanical behavior and network response to hydration. Rheological measurements were performed at room temperature under ambient light, using an angular frequency of 10 rad·s^–1^ and an oscillation strain of 0.1% to ensure testing within the linear viscoelastic region (LVR). The values of the rheological parameters for the linear viscoelastic region are compiled in Table 2.

**Table 2:** Rheology Data of Gel-1 and Gel-2 at Dry and Swollen States in the Linear Viscoelastic Region^a^

	*G*’ (kPa)	*G*” (kPa)	Tan δ (−)	η* LVR (kPa·s)	*η*’ (kPa·s)
Gel-1	5.2 ± 0.2	1.25 ± 0.01	0.241 ± 0.008	0.53 ± 0.02	0.12 ± 0.01
Gel-2	49.4 ± 0.8	33.89 ± 0.33	0.684 ± 0.003	6.01 ± 0.07	3.39 ± 0.03
Gel-1*	1.96 ± 0.05	0.022 ± 0.007	0.011 ± 0.004	0.196 ± 0.005	0.002 ± 0.0001
Gel-2*	0.34 ± 0.06	0.008 ± 0.002	0.026 ± 0.008	0.034 ± 0.006	0.001 ± 0.0001

a Swollen state.

In the dry state, Gel-2 exhibited significantly higher mechanical strength across all measured parameters compared to Gel-1 (Table 2). The storage modulus (*G*’) of Gel-2 reached 49.4 kPa, indicating a much stiffer and more elastic network, while Gel-1 recorded 5.2 kPa, suggesting a softer and more flexible structure. However, both gels were formulated with the same molar amount of EGDMA crosslinker; therefore, the higher stiffness of Gel-2 arises from structural contributions of the photoacid monomer. The increased E-MCMAH^+^ content not only introduces additional physical interactions, particularly hydrogen bonding and electrostatic attractions between protonated merocyanine and OEGMA chains, but also modulates local polymer segment dynamics, effectively restricting chain mobility. Such interactions increase the effective crosslink density, resulting in a stiffer, more viscous network even though the chemical crosslinking level remains constant. Furthermore, the bulky chromophoric side groups can enhance chain packing and micro-domain organization, contributing to higher rigidity in the dry state. Comparable reinforcement through noncovalent interactions has been observed in other methacrylate and ionic hydrogel systems, in which hydrogen bonding or ionic associations markedly increase modulus and mechanical stability.^34,35^ Upon hydration, these secondary interactions are disrupted, explaining the pronounced softening of Gel-2 in the swollen state. Nevertheless, the complex viscosity (η* LVR) of Gel-2 remained an order of magnitude higher than that of Gel-1 (6.01 kPa·s and 0.53 kPa·s, respectively). Furthermore, Gel-2 showed better damping properties and stronger dissipation of deformation energy, as confirmed by a much higher loss modulus (*G*”) of 33.89 kPa, compared to 1.25 kPa for Gel-1. The loss factor (tan δ) was also elevated in Gel-2 (0.684) relative to Gel-1 (0.241), additionally confirming that Gel-2 dissipates more deformation energy, reflecting a higher viscous contribution while still remaining in the predominantly elastic regime (tan δ < 1). Overall, Gel-2 possesses more rigid behavior, while Gel-1 maintains more elastic and compliant behavior, desirable for dynamic and responsive applications. The dynamic viscosity (η’) of Gel-2 was found to be 3.39 kPa·s, significantly higher than that of Gel-1 (0.12 kPa·s), thus Gel-2 characterizes better resistance to continuous deformation under steady shear. The elevated η’ in Gel-2 reflects increased energy dissipation and internal friction within the network, contributing to its superior mechanical rigidity. In contrast, the lower η’ value in Gel-1 suggests a more compliant network with easier flow, consistent with its softer and more elastic behavior. Analysis of viscoelastic properties of Gel-1 and Gel-2 as a function of frequency (Figure 4) confirmed the higher mechanical strength, enhanced stiffness, and better damping properties of Gel-2. For Gel-1, much lower values of *G*’ and *G*” were observed across the entire studied frequency range. A similar trend was detected for η’ values of both gels.

**Figure 4:**
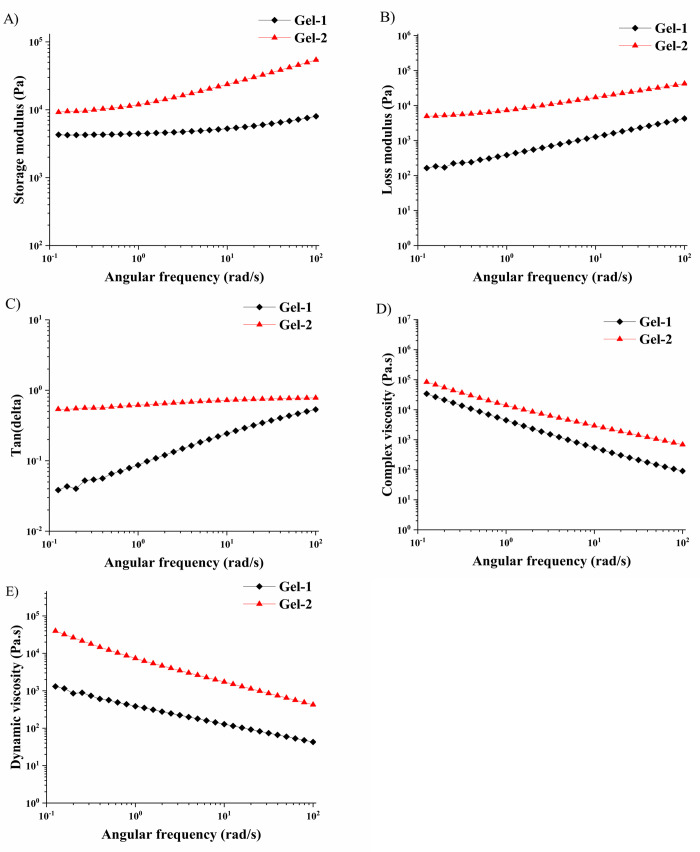
Rheology parameters of Gel-1 and Gel-2 in the dry state as a function of the frequency of deformation.

Upon swelling in water at pH 7, the two gels displayed distinct mechanical responses, which closely mirror their swelling behaviors (Table 2). Both gels showed a notable decrease in functional elasticity upon hydration, a unique and advantageous feature, visible as a decrease of *G*’. For Gel-1, the *G*’ value decreased to 1.96 kPa. In contrast, Gel-2 experienced a dramatic decrease in all of the rheological parameters after swelling. The *G*’ dropped significantly down to 0.34 kPa, and *G″* was reduced to 0.008 kPa·s, indicating structural weakening and collapse of mechanical integrity. The tan δ value in the swollen state for both gels decreased. The lower values of tan δ in the swollen state for Gel-1 suggest minimal energy dissipation and excellent recovery properties, likely arising from water-mediated hydrogen bonding and flexible interactions between the OEGMA segments and the surrounding solvent. These observations are well-aligned with the swelling behavior of Gel-1, which showed a swelling ratio of 1420%, along with physical thickness expansion from 0.960 mm to 2.280 mm. The improved elasticity in the swollen state supports the idea of a flexible yet stable polymer network capable of accommodating large volumes of water without mechanical failure. For both gels, the complex viscosity after swelling decreased but remained consistent with a structurally stable gel. η’ for both gels dropped significantly to values less than 0.002 kPa·s, supporting the minimal internal resistance to flow in the hydrated state. This softening behavior may be attributed to oversaturation of the network with photoacid functionalities, potentially leading to poor cross-link uniformity, which is typical for free radical polymerization, excessive water uptake, and matrix destabilization.^36,37^ Correlating with its lower swelling ratios (770%), Gel-2 appears to suffer from structural dilution upon hydration, highlighting the importance of optimizing functional monomer loading for mechanical robustness.

Similarly, the viscoelastic properties of swollen gels were studied as a function of frequency (Figure 5). The analysis confirmed a stronger reduction of *G*’ after swelling for Gel-2. The differences in the values of the loss shear modulus and the shape of the *G*″ plot as a function of frequency for both gels are connected with the differences caused by the varied content of photoacid, which could further affect the network structure. Due to the higher content of the hydrophobic photoacid, the liquid that is trapped can be easily expelled from the gel during measurement, leading to a stronger effect on the values of *G″* modulus, especially at low frequency. Different dissipation of energy due to the presence of water in the network also influenced the determined values of tan δ as frequency was changed. Significantly lower values of η* and η’ across the whole studied frequency range were determined for the swollen Gel-2.

**Figure 5:**
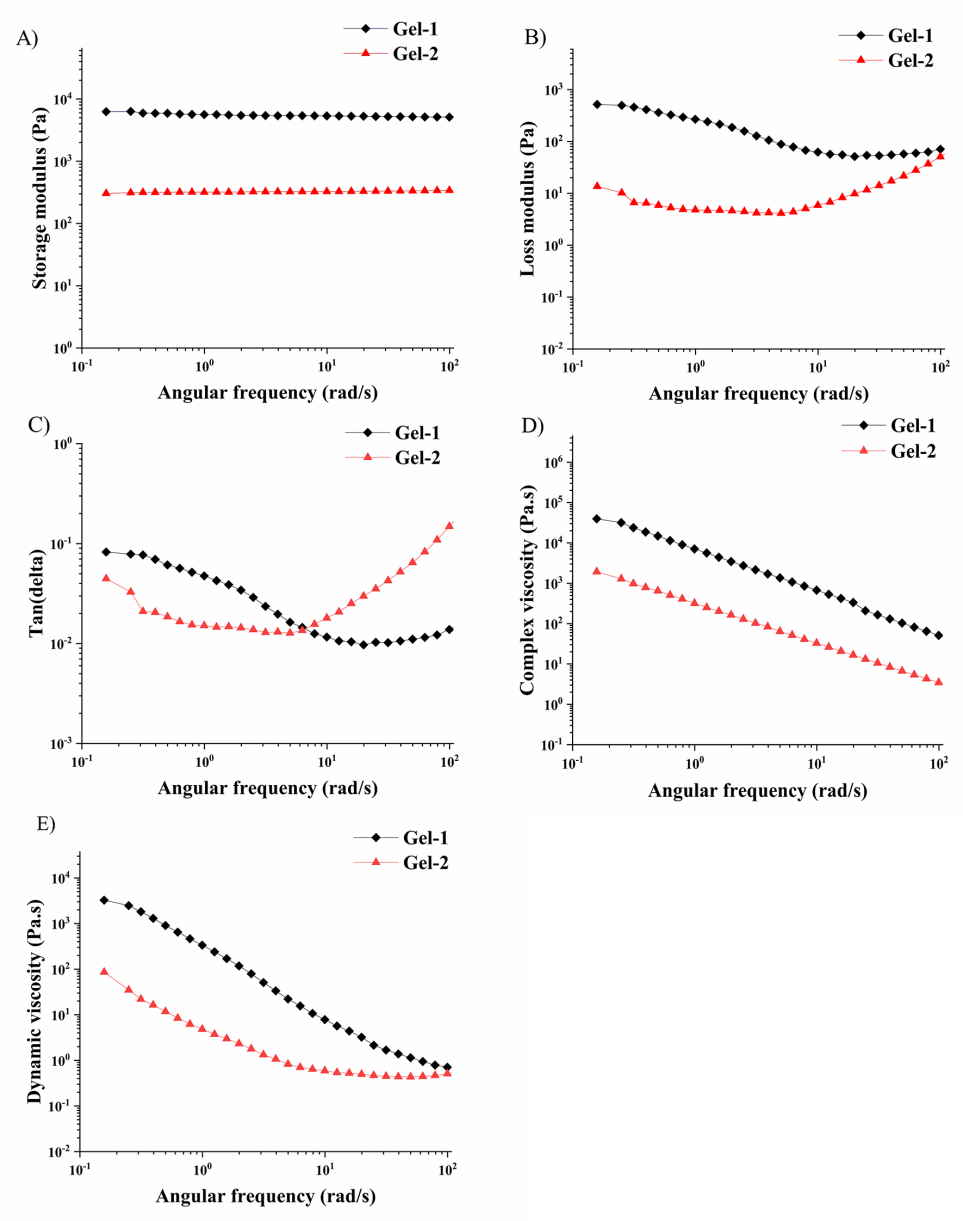
Rheology parameters of Gel-1 and Gel-2 in the swollen state as a function of the frequency of deformation.

Rheology data showed that Gel-2 was stiffer in the dry state but lost more mechanical strength upon swelling, while Gel-1 maintained better elasticity and resilience. This balance of flexibility and stability in Gel-1 is essential for biomedical use, where hydrated gels must remain mechanically robust. Based on these findings, Gel-1 was selected for further investigation.

### 3.4 Confirmation of the Gels’ Photoactivity System by Using Tauc’s Equation

Solid-state UV–vis spectroscopy was employed to investigate the optical behavior of the photoresponsive Gel-1 (Figure 6A). The spectra were recorded using a UV–vis spectrometer over a wavelength range of 200–1000 nm under both dark and 460 nm light-irradiated conditions for (i) POEGMA gel (Gel-0), (ii) P­(OEGMA-co-E-MCMAH^+^) gel in its initial form (Gel-1 in E-MCMAH^+^ form), (iii) P­(OEGMA-co-E-MCMAH^+^) gel after photochemical transformation of E-MCMAH^+^ to SPMA (Gel-1 in SPMA form), and (iv) recovered P­(OEGMA-co-E-MCMAH^+^) gel (Gel-1 in E-MCMAH^+^ form (reversed)). The observed spectral differences clearly reflect the distinct molecular and electronic environments of each state, and their transitions correlate well with the respective bandgap energies derived via Tauc plot analysis.

**Figure 6:**
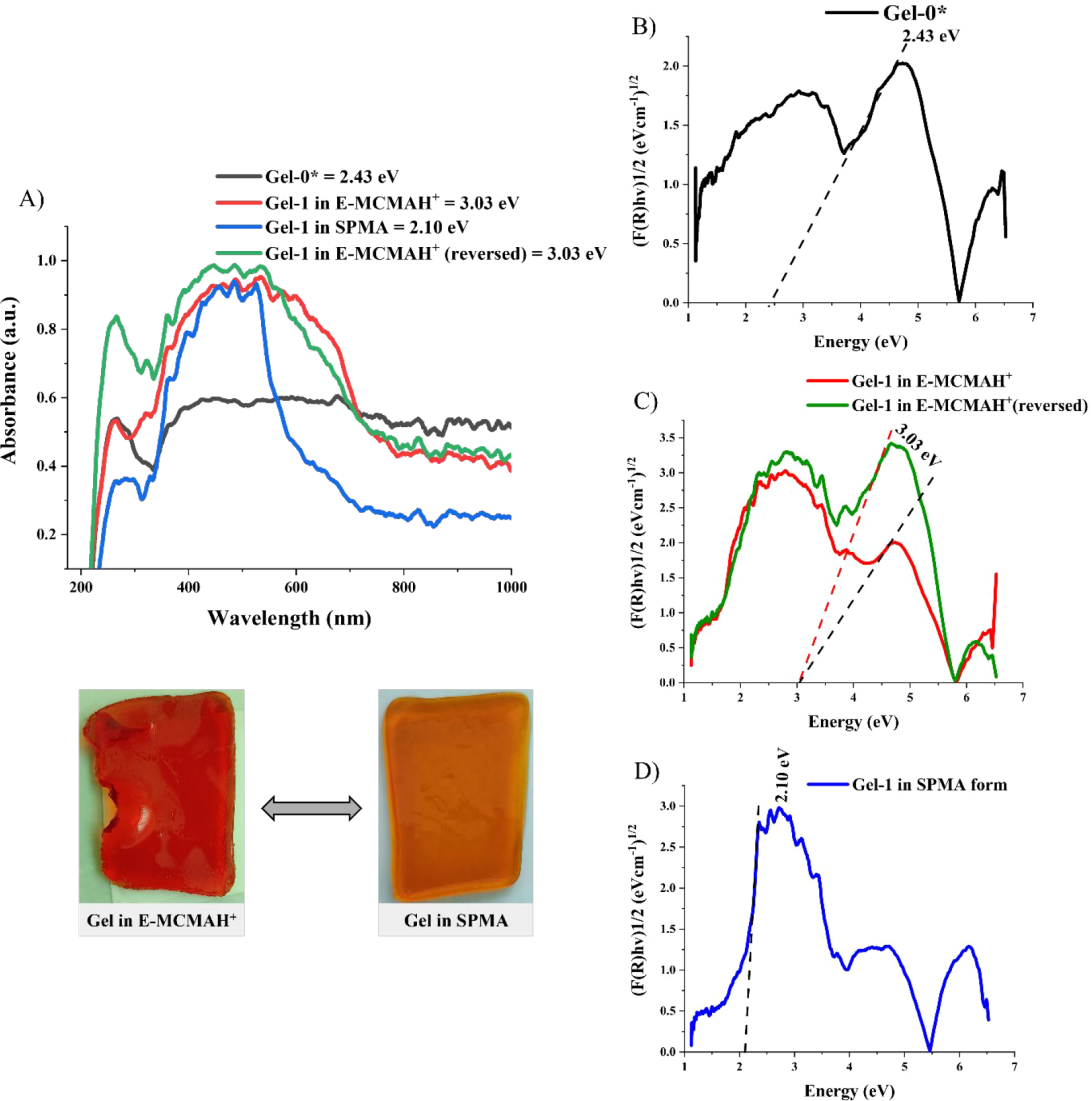
(A) Solid-state UV–vis spectra of the POEGMA-based gels. (B) Direct energy band gap calculated from Tauc’s plot for Gel-0. (C) Direct energy band gap calculated for Gel-1 with the initial merocyanine form and after the complete cycle of irradiation. (D) Direct energy band gap of Gel-1 with the spiropyran form.

The absorbance spectrum of the POEGMA control gel shows a relatively broad UV absorption peak between 280 and 350 nm, with a gentle decrease extending into the visible region (Figure 6A, black curve). This behavior is attributed primarily to n−π* transitions localized on the ester carbonyl groups in the methacrylate backbone and weakly delocalized electrons in the ethylene glycol side chains.^38^ As the polymer lacks extended aromatic conjugation, π–π* transitions are minimal. As shown by Tauc’s plot, assuming a direct allowed transition, the band gap was 2.43 eV (Figure 6B). This indicates a moderately wide optical gap, reflecting the nonconjugated structure.

The merocyanine-containing gel P­(OEGMA-co-E-MCMAH^+^) in its initial (dark) state, exhibited a pronounced absorbance peak at ∼315 nm, corresponding to π–π* transitions across the conjugated donor–acceptor system of the open merocyanine form (Figure 6A, red curve). Such a form exists as a zwitterion, with charge separated between indolinium nitrogen and phenolate oxygen, stabilized by intramolecular H-bonding and polar environments. Furthermore, this form is stabilized by the hydrophilic polymer environment.^39^ Figure 6C illustrates the direct bandgap derived for the merocyanine initial stage and after completion of the photocycle into its merocyanine form, derived as 3.03 eV, which is a conjugated but electronically localized chromophore that absorbs in the UV region.

Upon irradiation with 460 nm blue light in the presence of DMSO, the gel underwent a photochemical transformation to its spiropyran form. As a result, the absorbance spectrum displayed a shifted profile, as shown in Figure 6A (blue curve), with a pronounced shoulder in the 430–470 nm range and a diminished intensity at ∼315 nm. This spectral evolution reflects the ring-closing reaction of merocyanine to the spirocyclic spiropyran structure, where the orthogonal conformation disrupts π-conjugation and introduces new n−π* transitions at lower energies. The spiro form disrupts the extended conjugation, resulting in an electronically decoupled system where the two aromatic rings are arranged orthogonally. Consequently, n−π* transitions dominate, and new nonbonding molecular orbitals emerge, lowering the energy of allowed transitions. Figure 6D shows Tauc’s derived bandgap for the spiropyran state, which reduced to approximately 2.10 eV, consistent with the presence of a lower-energy, mixed electronic transition profile.

To assess the reversibility of this photochromic transformation, the spiropyran gel was protonated by using aqueous 0.01 mM HCl and exposed to UV light (365 nm). This promoted ring-opening and regeneration of the merocyanine form through acid-gated photochromism, where protonation lowers the thermal barrier for ring-opening. The spectrum shown in Figure 6A (green curve) for the gel reconverted to the merocyanine form closely resembles that of the original merocyanine-containing gel, with restored absorbance features at approximately 315 nm and an absorption edge near 420 nm. The calculated bandgap remained at 3.03 eV (Figure 6C), confirming the efficient recovery of the electronic structure of the merocyanine form after cycling through spiropyran.

The spectral and bandgap data across all states reveal important insights into the structure–property relationships within the gel matrix. The higher bandgap of the merocyanine state reflects the localized π-system, while the lower bandgap of spiropyran arises from new electronic transitions associated with its closed, n−π*-rich structure. Notably, the POEGMA matrix contributes only weakly to the absorbance profile, confirming that the spectral changes are primarily due to the dynamic behavior of the photoacid units. Moreover, the retention of spectral features and bandgap values across switching cycles confirms the gel’s robust photoreversibility under solid-state conditions. This reversible modulation of the electronic structure, governed by light and pH stimuli, highlights the potential of the gel system as a functional material capable of light-triggered proton release. The ability to switch between optically and electronically distinct states in a controlled, nondestructive manner opens promising avenues for its application in environments requiring a tunable pH response, particularly for advanced wound dressings. The use of visible light (460 nm) rather than harmful UV light also ensures biocompatibility. The strong UV absorbance by the gel components (notably between 300 and 350 nm) further contributes a protective function by attenuating potentially damaging UV radiation, which can otherwise lead to reactive oxygen species formation and impair tissue regeneration.

### 3.5 Light-Induced pH Modulation and Quantitative Proton Release from Merocyanine-Functionalized Hydrogels

To investigate the light-responsive behavior of the photoacid units, the E-MCMAH^+^ form was subjected to blue LED irradiation, triggering its transformation into the SPMA form via proton-releasing photoisomerization pathways (Figure 7A).^13,15,16,18^ Therefore, the photoresponsive behavior of the E-MCMAH^+^ containing hydrogels was investigated by quantifying the extent of pH changes in aqueous media under blue light irradiation (460 nm,14 mW/cm^2^) over a period of 1 h vs the expected theoretical pH drop. This experiment was conducted in Milli-Q water to evaluate the intrinsic proton-release behavior of the methacrylate-based moieties within the hydrogel network. Conducting the experiments in Milli-Q water, therefore, allows a direct and accurate assessment of the gel’s fundamental light-induced proton-release capacity, providing a clear picture of its intrinsic photoacidic behavior.^40^ A focused approach was taken on embedded photoacids, which affect the efficiency of light-triggered proton release. Gel-1, weighing 181 mg and containing 6.25 mol % of E-MCMAH^+^, was submerged in 20 mL of deionized water and irradiated under controlled conditions. The purpose of this design was to determine whether a higher absolute quantity of photoacid could enhance the magnitude of environmental acidification, independent of the relative concentration.

**Figure 7:**
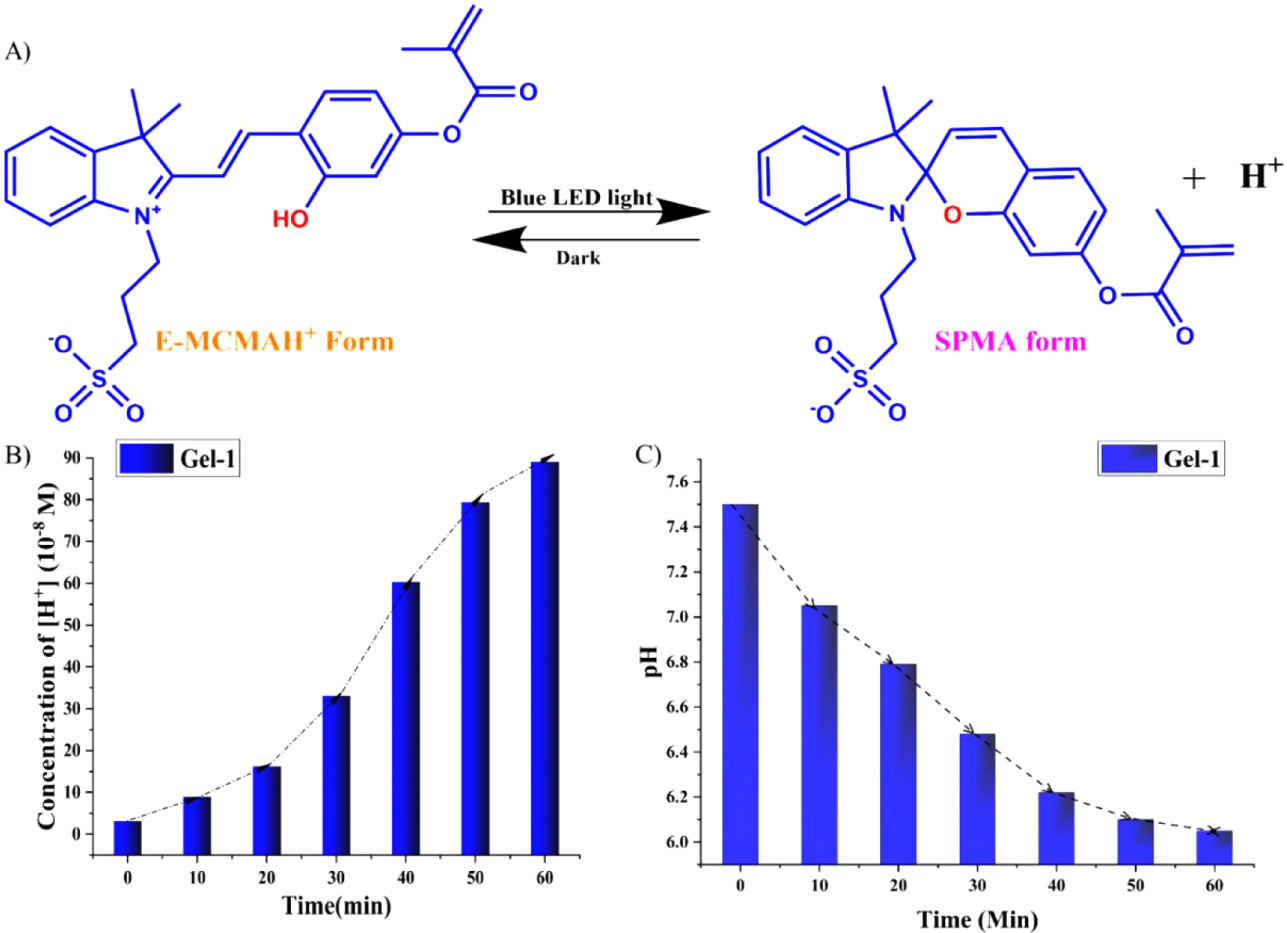
(A) Mechanism of transition of the E-MCMAH^+^ form to the SPMA form. (B) Experimental and theoretical profiles of acid-release concentration expressed as [H^+^] for Gel-1. (C) Corresponding experimental and theoretical pH drop module for Gel-1.

Irradiation of Gel-1 induced a progressive release of H^+^ ions over time (Figure 7B), which, in turn, resulted in a marked decrease in pH from 7.5 to 6.05 (ΔpH = 1.45) (Figure 7C). Using the pH change and the known buffer-free water volume (20 mL), the net concentration of protons released was calculated to be approximately 2.82 × 10^–6^ mol H^+^. Theoretical calculations were employed to estimate the maximum possible pH drop, providing a benchmark for comparison with the experimentally observed values (referred to in SI Section 3.7). These calculations indicate that complete conversion of all photoacid molecules within the hydrogel would yield proton concentrations on the order of 1.1 × 10^– 3^ M in the 20 mL aqueous solution, corresponding to a pH near 2.95. However, the experimentally observed pH drop was significantly smaller, reflecting the inherent limitations in the system, including incomplete photoconversion, restricted proton diffusion from the gel matrix into the bulk solution, and the buffering effect of water’s autoionization.

Nevertheless, this moderate but reversible acidification is sufficient to modulate the local microenvironment at the hydrogel surface. Small pH variations (≈1 unit) are known to influence bacterial metabolic activity and membrane potential, thereby providing a nonbiocidal yet inhibitory effect that supports the hydrogel’s function as an antibacterial wound dressing. The ability to achieve controllable, localized pH modulation rather than large bulk acidification is central to the intended functionality of this photoacid-based material.^41,42^

### 3.6 Photoacid-Induced pH Modulation Monitored via Electrochemical Techniques

To further investigate the pH-responsive behavior of the merocyanine containing gel, cyclic voltammetry (CV) and differential pulse voltammetry (DPV) were conducted. Oxidation of E-MCMAH^+^ within the hydrogel proceeds through a proton-coupled electron transfer (PCET) mechanism, where the deprotonated phenolic group (phenolate) undergoes electron loss to generate a phenoxyl radical, with further oxidation leading toward quinone-like structures. Protonation of the phenolic oxygen stabilizes the oxidized form, shifting the potential positively, whereas deprotonation lowers the oxidation potential.^43,44^ Reduction corresponds to electron addition to the conjugated merocyanine π-system, which redistributes electron density along the polymethine chain and alters the protonation state of the phenolic group. These processes are favored when the π-system is extended and electron-deficient.^43,44^ Within the hydrogel matrix, restricted proton mobility and local dielectric effects modulate the redox response, while light-induced E-MCMAH^+^ → SPMA conversion further increases the electroactive population and proton activity, producing characteristic shifts in CV and DPV profiles.^44–47^ The Gel-1 was subjected to light irradiation to induce the photoisomerization of the E-MCMAH^+^ form to the SPMA form, leading to proton release, which is a central functional output of these hydrogels. It was of interest to quantify the extent of light-induced local pH changes along with the evaluation of the accompanying changes in swelling behavior (Figures S4 and **S5**). The electrochemical measurements were performed by using the gel-coated platinum wire electrode and immersing it in two different solvent systems: (i) aprotic DMSO and (ii) aqueous 0.1 M KCl. For each solvent, electrochemical profiles were recorded under dark conditions and then again after 10 min of visible light irradiation (referred to as T10).

To quantify the pH drop from the redox potential, the underlying principle linking the electrochemical data to pH change lies in PCET mechanisms. The redox potential (*E*) of such systems varies linearly with pH and is described by the Nernst equation:

1 $$E=E^{0}-\frac{0.0591}{n}\cdot \log\left(\frac{\mathrm{Red}}{\mathrm{Ox}}\right)-\frac{0.0591\cdot m}{n}\cdot \mathrm{pH}$$

Where: *E* is the observed electrode potential; *E* ^0^ is the standard potential; *n* is the number of electrons; and *m* is the number of protons. When *m* = *n* = 1, the slope simplifies to approximately −59.1 mV per pH unit.

Hence, ΔpH = −Δ*E*_peak_/0.0591, thus this inverse proportionality between peak potential and pH enables the calculation of the local pH shift based on the electrochemical data (Table 3). However, the sensitivity and resolution of CV and DPV play a role in detecting pH-dependent redox processes. DPV is inherently more sensitive and better suited for analyzing subtle changes, such as local pH variation, while CV provides broad information about redox behavior and is highly useful for qualitative analysis.^47,48^ The continuous potential sweep leads to capacitive current contributions and peak broadening; thus, this shows the decrease in resolution and accuracy in determining the precise peak potentials.

**Table 3:** pH Module with Respect to the CV and DPV Peak Shifts in DMSO and in 0.1 M KCl

	CV reduction	DPV reduction	CV oxidation	DPV oxidation
DMSO				
Δ*E*	+40 mV	+50 mV	+60 mV	+80 mV
ΔpH	–0.68	–0.85	–1.01	–1.35
0.1 M KCl				
Δ*E*	+60 mV	+70 mV	+80 mV	+90 mV
ΔpH	–1.01	–1.18	–1.35	–1.52

The pH drop values calculated from CV voltammograms (Figure S4A and B) and DPV voltammograms (Figure S5A and B) in DMSO indicated that the gel releases protons under irradiation even in aprotic media, although the effect is modest due to limited proton mobility. This observation suggests that, although bulk proton transport in DMSO is slow, the photogenerated protons remain in a long-lived dissociated state at the hydrogel-electrode interface before recombining, allowing them to accumulate locally and shift the redox equilibrium within the short irradiation period. On the other hand, the pH drop values calculated from CV voltammograms (Figure S4C and D) and DPV voltammograms (Figure S5C and D) in 0.1 M KCl indicated that shifts are more pronounced in aqueous media due to enhanced proton conductivity and diffusion, leading to more efficient detection of the localized acidification values. This is because the polar environment facilitates faster proton exchange and equilibration between the hydrogel surface and the surrounding electrolyte, producing greater interfacial acidification signals.

Apart from the peak shifts, changes in peak current also offer qualitative insight into the proton dynamics; i.e., the oxidation currents increased due to enhanced PCET, or reduction currents were encountered by the acid suppression of the redox species. These current changes further confirm that the proton release process is confined primarily to the interface, where concentrations can be substantially higher than those in the bulk, enabling more pronounced effects to be detected electrochemically. Thus, these changes reinforce the conclusion that protons are released upon light exposure, and the resultant environment becomes more acidic. Electrochemical measurements revealed a pH drop of 1.0–1.5 units within just 10 min of irradiation. This discrepancy can be attributed to the fact that electrochemical techniques detect immediate, localized proton concentrations at the gel–electrode interface rather than the averaged pH of the bulk solution. In these confined microenvironments, proton accumulation occurs more rapidly and to a greater extent. Although normal pH measurements and electrochemical data ultimately converge, the key differences lie in the irradiation duration and the measurement methodology employed. Thus, electrochemical techniques detect immediate, localized proton concentrations at the electrode interface, where proton accumulation is the highest due to confinement and gel proximity. The electrochemical characterization shows slightly higher sensitivity and spatial resolution with respect to the light irradiation duration, although it enables real-time monitoring of dynamic changes occurring in microenvironments, which is critical for applications where local pH plays a functional role.

Electrochemical monitoring of Gel-1 under light irradiation revealed a distinct and reproducible decrease in solution pH, confirming that the light-triggered proton release is directly associated with the merocyanine-to-spiropyran conversion. The sharp and controlled pH drop demonstrated efficient proton liberation, while the stability of the response over repeated cycles reflected the photochemical robustness of the gel. These results not only validated the functional activity observed in the solid-state optical studies but also provided quantitative evidence of its operational capacity in aqueous environments.

### 3.7 *In Vitro* Cytotoxicity and Phototoxicity of Photoresponsive Gel

The cytotoxicity test, conducted according to the ISO 10993-5 standard for medical devices, revealed that the gel was noncytotoxic since cell viability was equal to 105% compared to the negative control (Figure S6). Direct cytotoxicity analysis using a confocal laser scanning microscope (CLSM) confirmed the nontoxic nature of the tested gel. Numerous viable cells (green fluorescence) were observed growing around the tested gel, displaying morphology comparable to that of control cells cultured on a polystyrene well (Figure 8). Importantly, no dead cells (red fluorescence) were observed. The high biocompatibility can be attributed to the gel’s composition. POEGMA is well-known for its low toxicity and protein-repellent properties, which minimize adverse cellular interactions. The presence of merocyanine did not change the character of the gel. The photoinduced E-MCMAH^+^-to-SPMA conversion, which releases protons, occurs only under 460 nm light, which was not applied during the cytotoxicity tests, ensuring that the gel remained in its nonactivated, biocompatible state.

**Figure 8:**
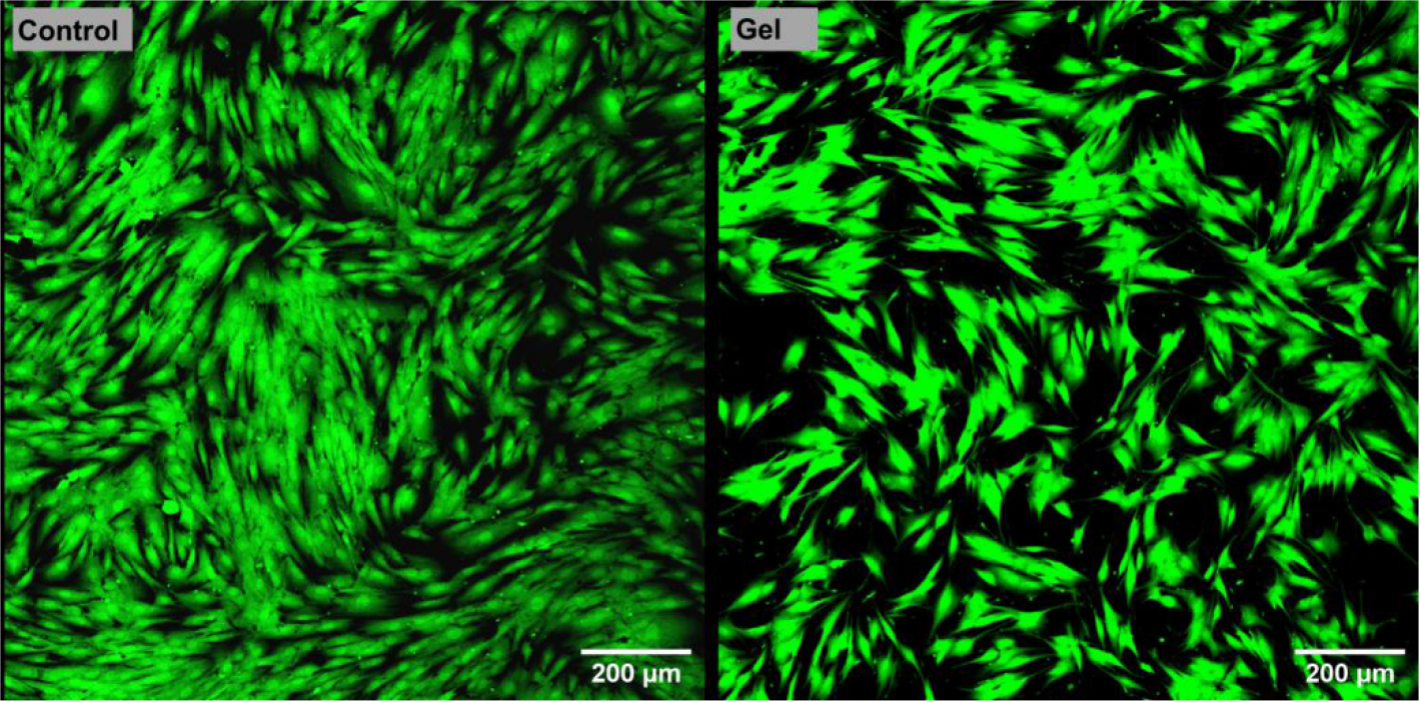
Confocal laser scanning microscope images showing live/dead staining of BJ cells cultured in the presence of the tested gel (control – cells growing on a polystyrene well).

Phototoxicity tests were performed to determine how exposure of the gel to 460 nm light affects cell viability. For this purpose, BJ cells were cultured in the presence of the tested gel and exposed to blue light. The test revealed a statistically significant decrease in cell viability after 2- and 3-h exposure to blue light (Table 4). Nevertheless, cell viability remained above 70%. Importantly, after the removal of the light source, the cells were cultured for 12 h in the dark, and a further decrease in cell viability was observed, indicating a cytotoxic effect. This phenomenon was most likely related to changes in the pH of the medium during sample irradiation. It is worth noting that *in vitro* cell cultures are very sensitive to pH changes. Although the physiological pH level of the skin is slightly acidic (from 4.5 to 6.0),^49^ it may significantly reduce fibroblast viability under *in vitro* conditions. Therefore, *in vitro* studies do not reflect the microenvironment of *in vivo* conditions during chronic wound healing. In turn, chronic wounds are characterized by an alkaline pH (often reaching 8.9), making them difficult to heal.^2,50^ Thus, gel irradiation causing a decrease in pH level may be beneficial under *in vivo* conditions during chronic wound treatment despite the observed reduction in cell viability *in vitro*. Lowering the wound pH will facilitate the healing process since pH < 7 provides optimal conditions for fibroblast proliferation *in vivo.* It is also crucial to note that in the analyzed system, cell viability was influenced not only by the gel itself but also by an additional factor, i.e., blue light. According to the available literature, exposure conditions to blue LED light can cause both beneficial photobiomodulation effects at low doses and potential stress or cytotoxic effects at high doses.^51,52^

**Table 4:** BJ Cell Viability after Exposure to Blue Light^a^

	Blue light (460 nm) exposure time		
**Viability**	2 h	3 h	3 h + overnight incubation
	79% ± 0.8%	72% ± 1.6%	55% ± 1.2%

a Statistically significant results compared to nonirradiated control determined by an unpaired *t*-test, *p* < 0.05.

### 3.8 Antimicrobial Test

Antibacterial properties were investigated first on *Pseudomonas aeruginosa*, which is a Gram-negative, opportunistic human pathogen and a common cause of hospital-acquired infections. Its pathogenicity relies on a broad repertoire of chemically diverse virulence factors, including toxins, proteases and small molecule metabolites.^53^ The laboratory strain PAO1, used for modeling a moderately virulent phenotype, has contributed significantly to facilitating the discovery of new drug targets and the testing of novel therapeutic approaches.^54,55^ Like other *Pseudomonads,* it shows a strong tendency to form structured, multicellular biofilms on abiotic and biotic surfaces (e.g., medical devices, wound tissues), conferring resistance to environmental stresses and antimicrobial treatment.^55^ These features make *P. aeruginosa* a paradigm for bacterial persistence and therapeutic recalcitrance and highlight the urgent need for fundamentally new treatment approaches. Methicillin-resistant *Staphylococcus aureus* (MRSA) represents another clinically highly relevant pathogen and a leading cause of community- and hospital-acquired infections of soft tissues.^56^ *S. aureus* is a Gram-positive bacterium characterized by its thick peptidoglycan cell wall and is known for its resistance to β-lactam antibiotics, including methicillin.^57^ MRSA can colonize the skin and wound tissues, where it can form persistent biofilms that protect the cells from host immune responses and antimicrobial treatment.^58,59^ Given its distinct cell envelope structure and resistance mechanisms, MRSA serves as a complementary model organism to *P. aeruginosa* for evaluating the antibacterial performance and mode of action of our photoacid-based hydrogels. In this context, advanced materials such as our proposed E-MCMAH^+^ contains gels are promising innovative approaches to wound care that can combat microbial infections.

As an initial control experiment, the effects of (acidic) pH and blue light irradiation on both bacterial strains in the absence of any hydrogel material were evaluated. Therefore, optical densities (OD at 600 nm) and cell viabilities were measured under neutral (pH 7.0) and acidic conditions (pH 5.8), either kept in the dark or exposed to blue light. At both neutral and acidic pH, blue light irradiation resulted in a significant reduction in cell densities and viable cells for both strains compared with the corresponding dark controls (Figure 9), confirming that light exposure alone already induces measurable physiological stress. Importantly, acidification alone caused only minor cell number and viability reductions in the dark, whereas the strongest effects occurred after irradiation (Figure 9B). These results show baseline sensitivities of both *P. aeruginosa* and MRSA to pH shifts and blue light and provide a necessary reference framework for interpreting the enhanced antimicrobial activity observed with the photoacid hydrogels.

**Figure 9:**
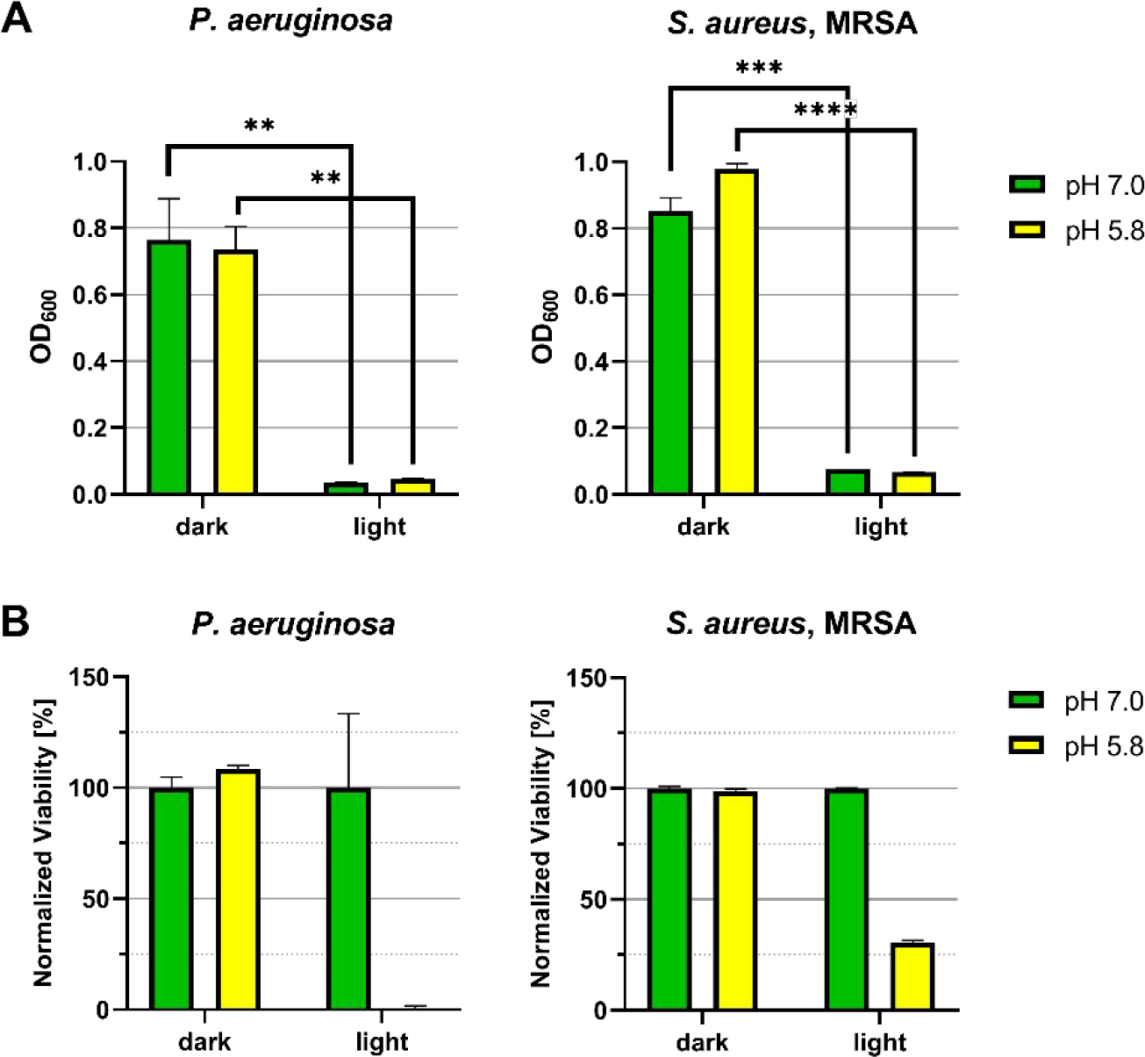
(A) Optical densities and (B) viabilities of *P. aeruginosa* and *S. aureus* MRSA under neutral (pH 7.0) or acidic (pH 5.8) conditions with and without blue light irradiation at 350 mA intensity. Statistical significance was determined by Student’s *t*-test and *p* < 0.05 were considered statistically significant. ** denotes *p* < 0.01, *** denotes *p* < 0.001, and **** denotes *p* < 0.0001.

To determine and quantify the antibacterial activity of Gel-1, a resazurin reduction assay was performed. Even without irradiation, the cell viability was greatly reduced (Figure 10), corresponding to ∼60% bactericidal activity in the dark for *P. aeruginosa* (Figure 10A). Upon blue light irradiation, the cell viabilities were completely reduced due to the inhibition of cell growth (Figure 9). The light source was employed to trigger the conversion of the E-MCMAH^+^ form into the SPMA closed-ring form. This light-driven ring-closing process is accompanied by proton release into the hydrogel microenvironment, which results in a localized and measurable drop in pH. Such proton release is highly effective against *P. aeruginosa*, as this pathogen is acutely sensitive to environmental acidification.^60,61^ MRSA is known for its higher resistance to lower pH environments, as it colonizes many acidic niches, including the host skin (pH 4.1–5.8),^62,63^ the vagina (pH 3.8–4.5)^64^ or biofilms in general.^65^ This was also observed here, as the reduction of cell viability without light irradiation of the hydrogels was not significant, at approximately 40% reduction (Figure 10B). However, after blue light exposure, the gels were perfectly effective and reduced the cell viability of MRSA completely (Figure 10B). Interestingly, despite structural differences in the cell envelopes of Gram-negative *P. aeruginosa* and Gram-positive MRSA, both species showed strong susceptibility to light-activated gels. This suggests that the antimicrobial mechanism is primarily governed by physicochemical processes, namely proton release and local acidification induced by the spiropyran-based photoacid, rather than species-specific molecular targets.

**Figure 10:**
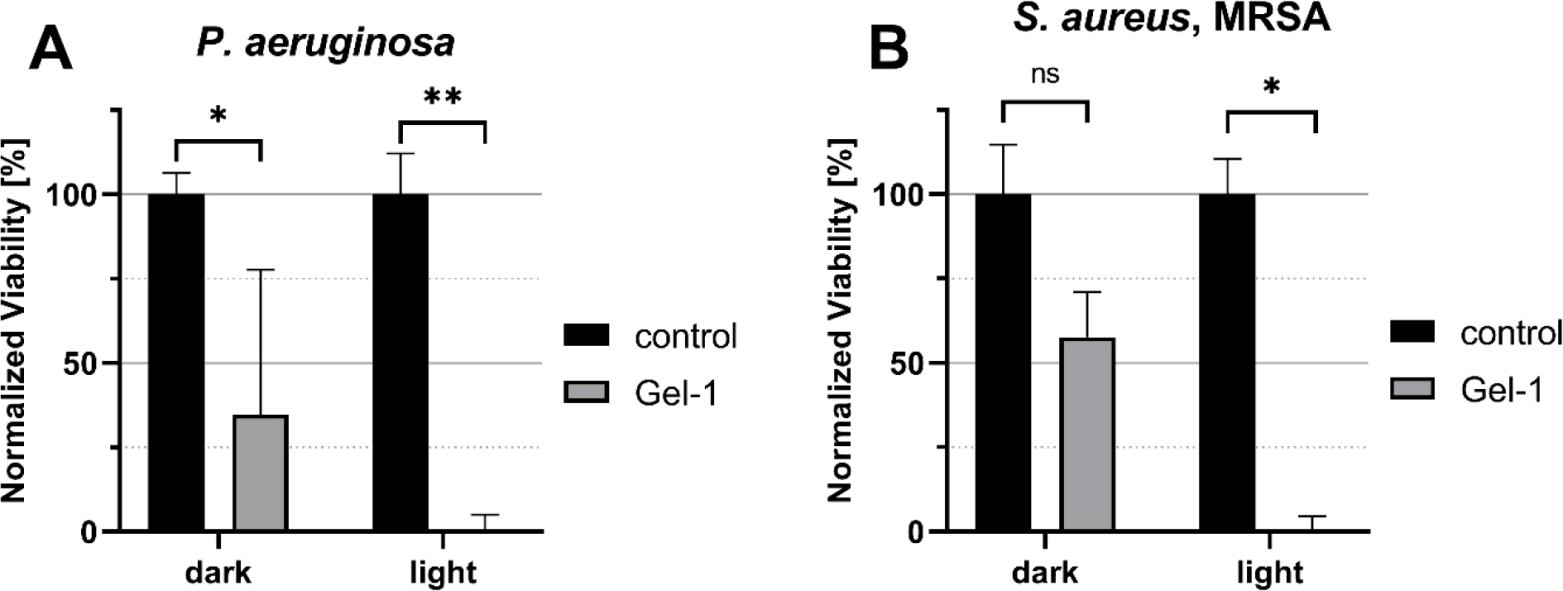
Viability reduction capability of Gel-1 after blue light irradiation at a 350 mA intensity. Normalized (A) *P. aeruginosa* PAO1 and (B) *S. aureus* MRSA viabilities after 24 h of incubation with Gel-1 or without gel as negative controls, determined in a resazurin reduction assay. Error bars symbolize standard deviations of measurements conducted in triplicates. Statistical significance was determined by Student’s *t*-test and *p* < 0.05 were considered statistically significant. * denotes *p* < 0.05, ** denotes *p* < 0.01, and ns denotes not significant.

Both the dark- and light-exposed hydrogels displayed notable antibacterial activity, which can be attributed to their intrinsic physicochemical mechanism of action. Upon blue-light irradiation, the spiropyran-based photoacid units within the gel undergo isomerization, releasing protons and thereby driving rapid local acidification. To further analyze whether proton release was accompanied by oxidative processes, the reactive oxygen species (ROS) formation was quantified using the Amplex Red assay, a sensitive fluorometric method for detecting hydrogen peroxide (H_2_O_2_) as a key marker of oxidative stress.^66,67^ Irradiated gels produced higher H_2_O_2_ levels compared to nonirradiated gel controls, confirming that light activation not only induces proton release but also promotes ROS generation in the surrounding medium (Figure 11). However, gels kept in the dark also showed certain ROS levels, consistent with their antibacterial potency (Figure 10).

**Figure 11:**
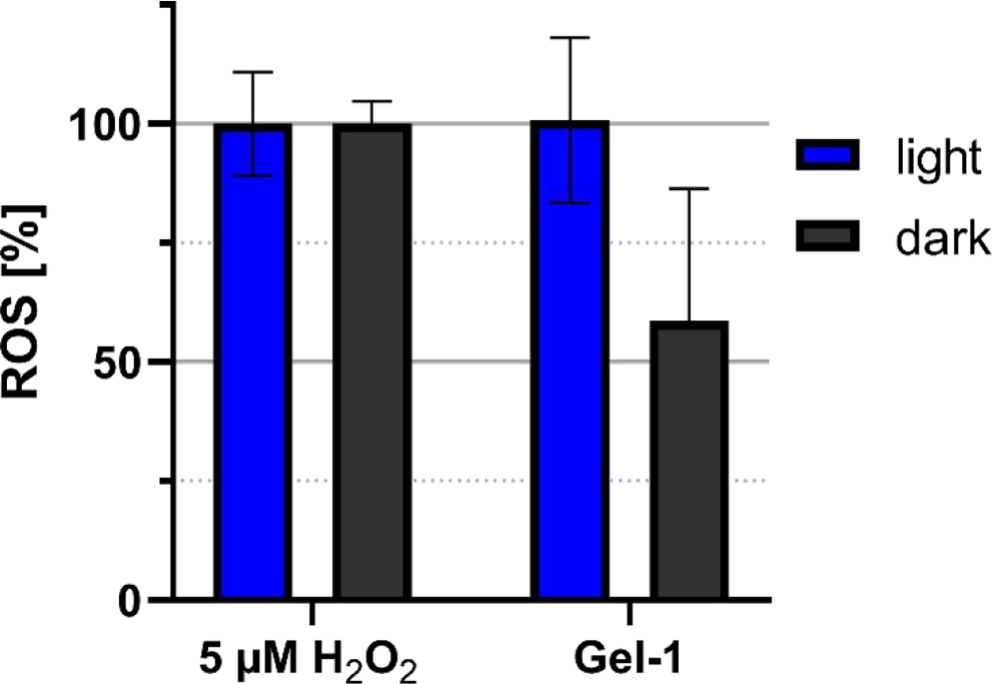
ROS production capability of Gel-1. Amplex Red fluorescence in response to 5 μM H_2_O_2_ and Gel-1 under dark- or blue-light-irradiated conditions. Error bars indicate standard deviations of experiments conducted in triplicate.

Together, these findings indicate that the enhanced antimicrobial efficacy of the photoacid hydrogels under blue light arises from a synergistic combination of proton release-driven acidification and light-induced ROS production, both of which contribute to the pronounced growth inhibition observed for *P. aeruginosa* and MRSA. Acidic stress is known to destabilize bacterial outer membrane proteins, interfere with proton motive force and energy metabolism, and disrupt quorum-sensing pathways that regulate virulence and biofilm formation. Consequently, the light-triggered E-MCMAH^+^ to SPMA conversion acts as a molecular switch that translates an optical stimulus into a chemical signal (acidification), thereby amplifying the antibacterial activity of Gel-1 beyond the level observed under dark conditions. These results highlight the potential of E-MCMAH^+^-based gels as dynamic, light-activated materials for controlling bacterial infections in the future.

## 4 Conclusions

In this study, merocyanine-based photoacids (E-MCMAH^+^) were successfully incorporated as comonomers with oligo­(ethylene glycol) methyl ether methacrylate (OEGMA) to synthesize multiresponsive hydrogels via free radical copolymerization. Syntheses were carried out in the dark to ensure the stability of the merocyanine form. The photoacid content within the gel system was optimized to achieve efficient proton release while maintaining favorable swelling behavior under both dark and light conditions. The viscoelastic properties of the hydrogels were enhanced, indicating improved structural integrity and stability. Solid-state UV spectroscopy, analyzed via Tauc’s equation, confirmed the photoisomerization of the merocyanine within the gel network. Furthermore, ionic transport and cyclic and differential pulse voltammetry studies demonstrated the electrochemical activity of the hydrogels and allowed quantification of proton release that closely matched theoretical predictions. The ability of the prepared hydrogels to effectively lower pH was confirmed, which is an essential feature for the healing of chronic wounds. Biological evaluations revealed that the gels were noncytotoxic under dark conditions, with BJ fibroblast viability exceeding 100%, and CLSM analysis confirmed healthy cell morphology and the absence of dead cells. Phototoxicity studies indicated a moderate decrease in cell viability under blue light exposure (460 nm), likely due to pH changes in the medium and light-induced stress. However, viability remained above 70% after 2–3 h, highlighting the potential for safe in vivo applications where localized pH reduction could promote wound healing. Antimicrobial assessments demonstrated that the hydrogels possess intrinsic antibacterial activity. Gel-1 reduced *Pseudomonas aeruginosa* viability by ∼60% in the dark and achieved complete bacterial eradication upon blue light irradiation, which triggers E-MCMAH^+^ to SPMA conversion and local acidification. MRSA, known for its tolerance to acidic environments, displayed moderate susceptibility in the dark (∼40% reduction), but complete inhibition was observed upon light activation. Light-triggered ROS generation further contributed to the antimicrobial efficacy. These results indicate that the antimicrobial mechanism is primarily governed by physicochemical processes, namely, local proton release and ROS production, rather than species-specific molecular targets. Overall, the prepared merocyanine-functionalized hydrogels exhibit a synergistic combination of controlled proton release, structural stability, and light-activated antimicrobial activity. These properties position them as promising smart materials for advanced wound dressing applications, capable of dynamically modulating the pH and combating pathogenic infections in a targeted and controllable manner.

## Supplementary Material



## Data Availability

The data will be available upon request.

## Notes

Detailed experimental procedures for E-MCMAH^+,^ quantifications of acid release studies for gels, digital microscopy evaluations of the gels, and figures of cyclic and differential pulse voltammetry (PDF)

## ○

Institut Européen des Membranes—IEM (UMR 5635), Univ Montpellier, CNRS, ENSCM, 34095 Montpellier, France
