# Supplementary material for: Photoactive Hydrogels Containing Merocyanine/Spiropyran Units for Wound Dressing Applications
Source: Biomacromolecules. 2025 Dec 29;27(1):684–701. doi: 10.1021/acs.biomac.5c01956 (PMC12801193; doi:10.1021/acs.biomac.5c01956)
Supplement: Supplementary file 1 [file bm5c01956_si_001.pdf]

Supplementary information

Photoactive Hydrogels Containing Merocyanine/Spiropyran Units For Wound Dressing Applications

Hitesh Katariya[1], Ewa Chrzescijanska[2], Krzysztof Jerczynski[1], Jan Rutkowski[1], Vishal Purohit[3], Magdalena Lipinska[1], Ahmet Cetinkaya[1X], Ann-Kathrin Kissmann[4], Daniel Gruber[4], Jan-Christoph Walter[4], Frank Rosenau[4], Aleksandra Maciejczyk[5], Agata Przekora[5], Jozef Kollár[6], Jaroslav Mosnáček[6,*], Joanna Pietrasik[1,*]

[1]Institute of Polymer and Dye Technology, Lodz University of Technology, Stefanowskiego 16, 90-537 Lodz, Poland

[X]Institut Européen des Membranes - IEM (UMR 5635), Univ Montpellier, CNRS, ENSCM, 34095 Montpellier, France (current address)

[2]Faculty of Chemistry, Institute of General and Ecological Chemistry, Lodz University of Technology, Zeromskiego 116, 90-924 Lodz, Poland

[3]Department of Chemical Sciences, P. D. Patel Institute of Applied Sciences, Charotar University of Science and Technology (CHARUSAT), Changa, 388 421, Gujarat, India

[4]Institute of Pharmaceutical Biotechnology, Ulm University, Meyerhofstrasse 1, 89081 Ulm, Germany

[5]Department of Tissue Engineering and Regenerative Medicine, Medical University of Lublin, Chodzki 1, 20-093 Lublin, Poland

[6]Polymer Institute of the Slovak Academy of Sciences, Dubravska cesta 9, 845 41 Bratislava, Slovakia

** Corresponding authors: jaroslav.mosnacek@savba.sk, joanna.pietrasik@p.lodz.pl*

1. **Synthesis of (E)-3-(2-(2-hydroxy-4-(methacryloyloxy)styryl)-3,3-dimethyl-3H-indol-1-ium-1-yl)propane-1-sulfonate (E-MCMAH$^+$)**

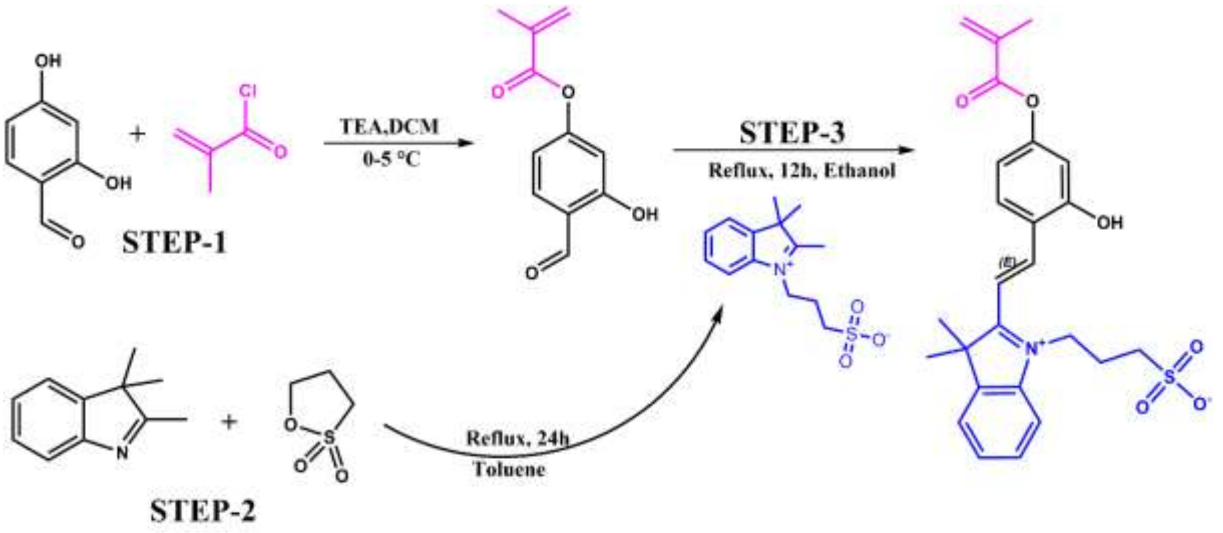

Figure S1. The scheme for synthesis of merocyanine based methacrylate photoacid.

Three-step procedure was applied (**Figure S1**)[1]. Step 1: 2,4-Dihydroxybenzaldehyde (10 g, 0.072 mol) was dissolved in 500 mL of methylene chloride while maintaining the temperature at 0-5 °C. Next, triethylamine (23.2 mL, 0.170 mol) was added, followed by the dropwise addition of a solution of methacryloyl chloride (8.8 mL, 0.090 mol) in 100 mL of methylene chloride over 30 minutes at 0-5 °C. The reaction was carried out at room temperature and monitored using thin layer chromatography (TLC). The mixture was washed 3 times with water, then the organic phase was separated, magnesium sulfate ($MgSO_4$) was used to remove residual water, and solvent was evaporated under the vacuum. The crude product was purified by column chromatography (n-hexane/ethyl acetate – 20/1) to obtain white solid in 85% yield.

Step 2: A solution of 2,3,3-trimethyl-3H-indole (7.1 mL, 0.044 mol) and 1,2-oxathiolane-2,2-dioxide (7.0 g, 0.057 mol) in 65 mL of toluene (65 mL) was refluxed overnight at 120 °C under an inert atmosphere. Products were obtained by washing with petroleum ether under vacuum filtration. A dry purple powder was obtained in 78% yield.

Step 3: The following solution was prepared; step 2 product (4.4 g, 0.016 mol) and step 1 product (5.0 g, 0.024 mol) in 65 mL of ethanol were stirred for 6 hours at 90 °C under inert atmosphere. After cooling, the precipitates were filtered and washed with cold ethanol several times under high vacuum filtration. The merocyanine based methacrylate photoacid (MCMA) was collected as a tangy orange solid in 72% yield. [^{1}H NMR (400 MHz, DMSO-d_6) (**Figure**

S2) δ 11.40 (s, 1H), 8.51 (d, 1H), 8.33 (d, 1H), 8.04 – 7.94 (m, 1H), 7.88 – 7.72 (m, 2H), 7.71 – 7.52 (m, 2H), 6.85 – 6.72 (m, 2H), 6.30 – 6.22 (m, 1H), 5.90 (dp,1H), 4.77 (t, 2H), 2.60 (dt, 2H), 2.20 -2.10 (m, 2H), 2.01 – 1.92 (m, 3H), 1.73 (s, 6H)].

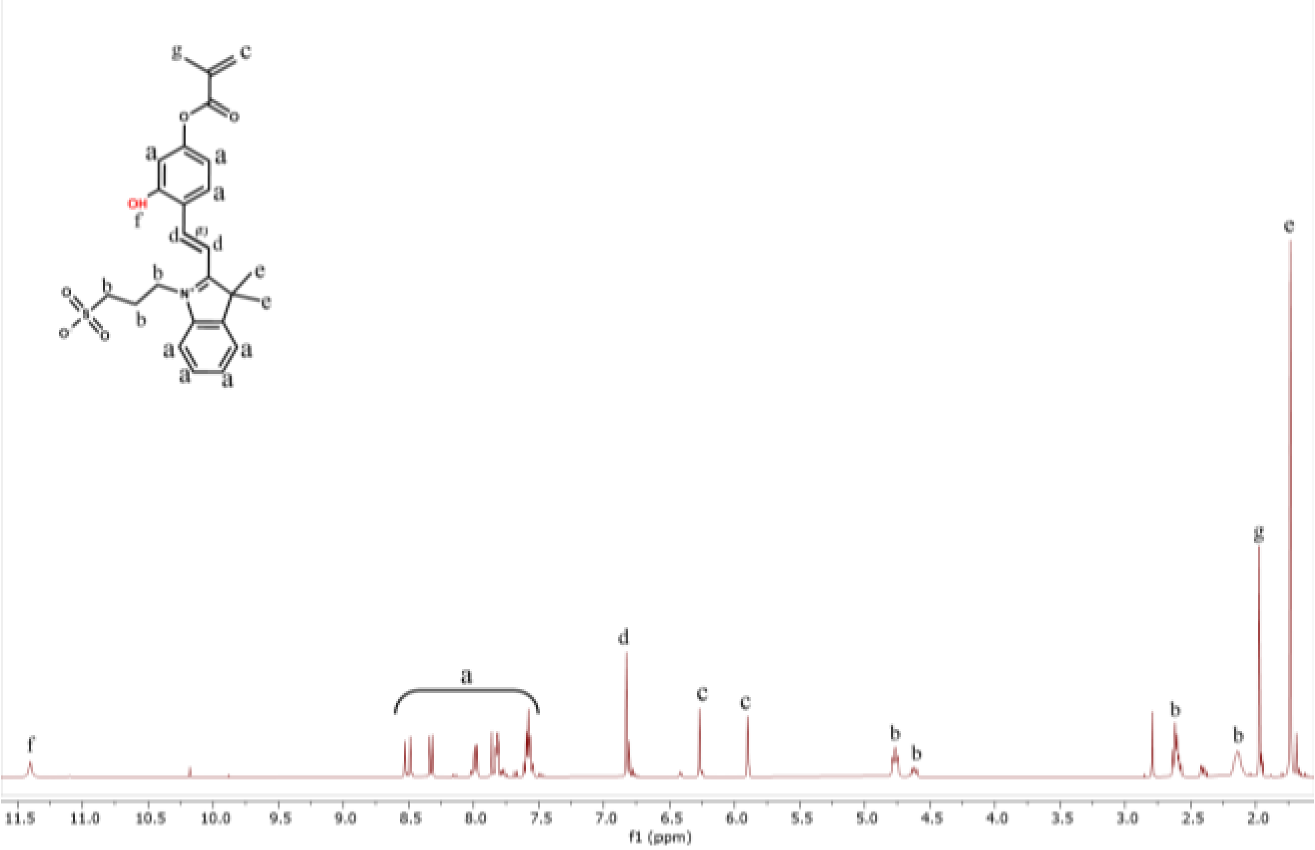

Figure S2. ^{1}H-NMR spectrum of the (E)-3-(2-(2-hydroxy-4-(methacryloyloxy)styryl)-3,3-dimethyl-3H-indol-1-ium-1-yl)propane-1-sulfonate.

2. Quantification of acid release from the gel

To quantify the concentration of the released protons due to the spiropyran formation after irradiation the pH changes of the water solutions were monitored. The pH is related to the proton concentration ($[H^+]$) by the equation:

$$\mathrm{pH} = -\log_{10}([H^+])$$

Rearranging this equation gives the proton concentration:

$$[H^+] = 10^{-pH}$$

For **Gel-1** (181.6 mg, 5.87 wt. % MC, in 20 mL DI water):

Initial pH:

$$7.5 \rightarrow [H^+]_{initial} = 3.16 \times 10^{-8} M$$

pH after irradiation:

$$5.9 \rightarrow [H^+]_{final} = 1.26 \times 10^{-6} M$$

Change in proton concentration:

$$\Delta[H^+] = 1.23 \times 10^{-6} M$$

Proton released (mol):

$$n[H^+] = \Delta[H^+] \times V = 1.23 \times 10^{-6} M \times 0.02\, L = 2.46 \times 10^{-8} mol$$

Mass of MC in gel:

$$5.87\% \times 181.6\, mg = 10.66\, mg$$

Moles of MC:

$$n_{MC} = \frac{10.66\, mg}{469.55 \frac{g}{mol}} = 2.27 \times 10^{-5} mol$$

Conversion efficiency:

$$\frac{n_{H^+}}{n_{MC}} \times 100 \approx 0.108\%$$

Theoretical pH drop calculations:

Total mass of photoacid in Gel-1 is 0.01011 g what corresponding to the total moles equal to 2.153 × 10^{-5} mol. Assuming 100% conversion, maximum moles of protons releases will be equal to moles of photoacid.

If all protons release evenly into 20 mL, proton concentration in solution for Gel-1 will be:

$$[H^+] = \frac{2.153 \times 10^{-5}}{0.02} = 1.0765 \times 10^{-3} M$$

Calculate theoretical pH from this proton concentrations

$$\text{pH} = -\log_{10}([H^+])$$

$$-\log_{10}(1.0765 \times 10^{-3}) = 2.97$$

A) B)

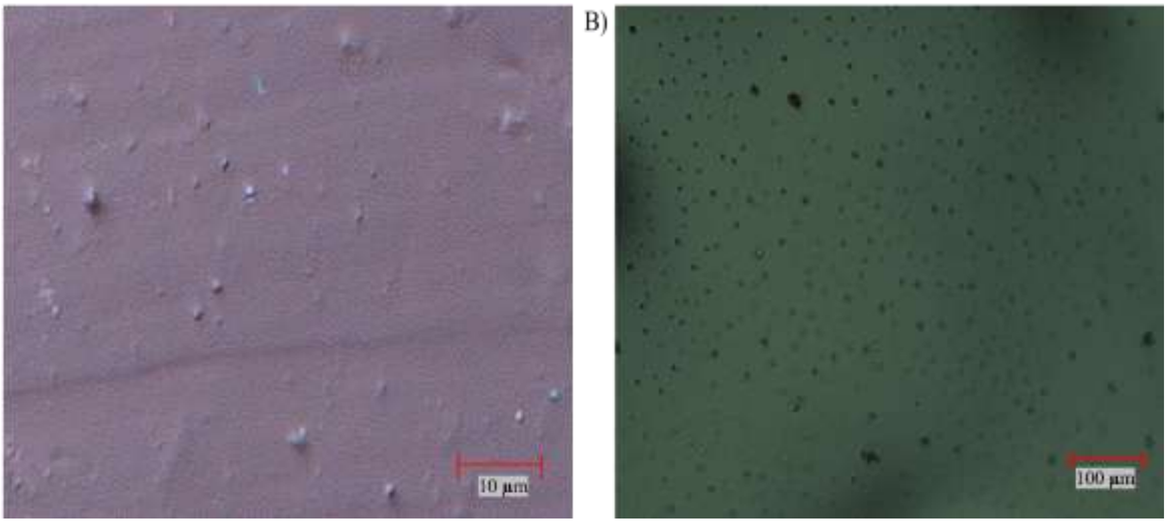

Figure S3. Digital microscopy pictures for (A) Gel-0 and (B) Gel-1.

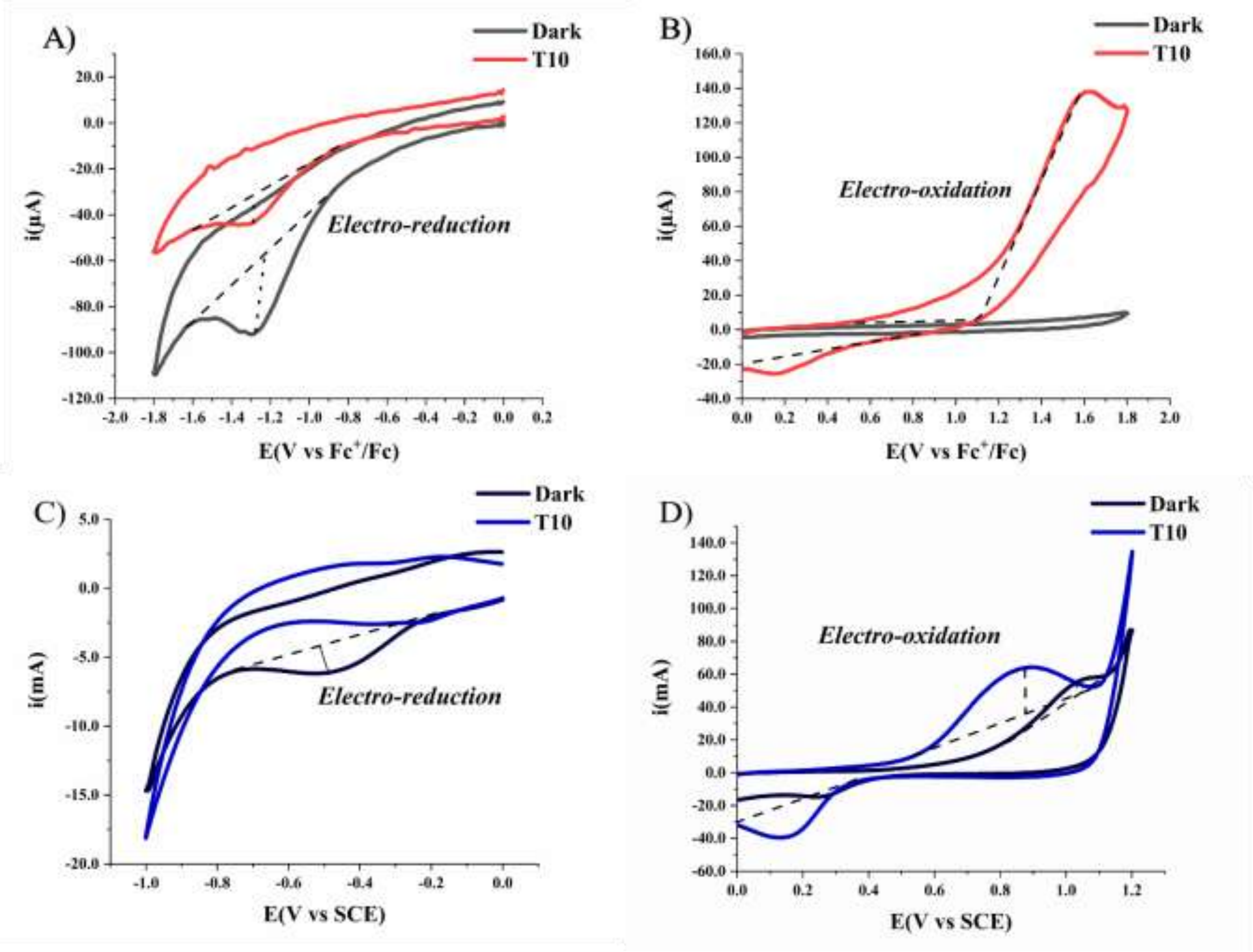

Figure S4. Representative cyclic voltammograms (CV) of Gel-1 recorded in dark and after 10 minutes of light irradiation (T10), (A) & (B) redox analysis at Pt electrode in 0.1 mol L^{-1} $(C_4H_9)_4NClO_4$ in DMSO and (C) & (D) redox analysis at Pt electrode in 0.1 M KCl.

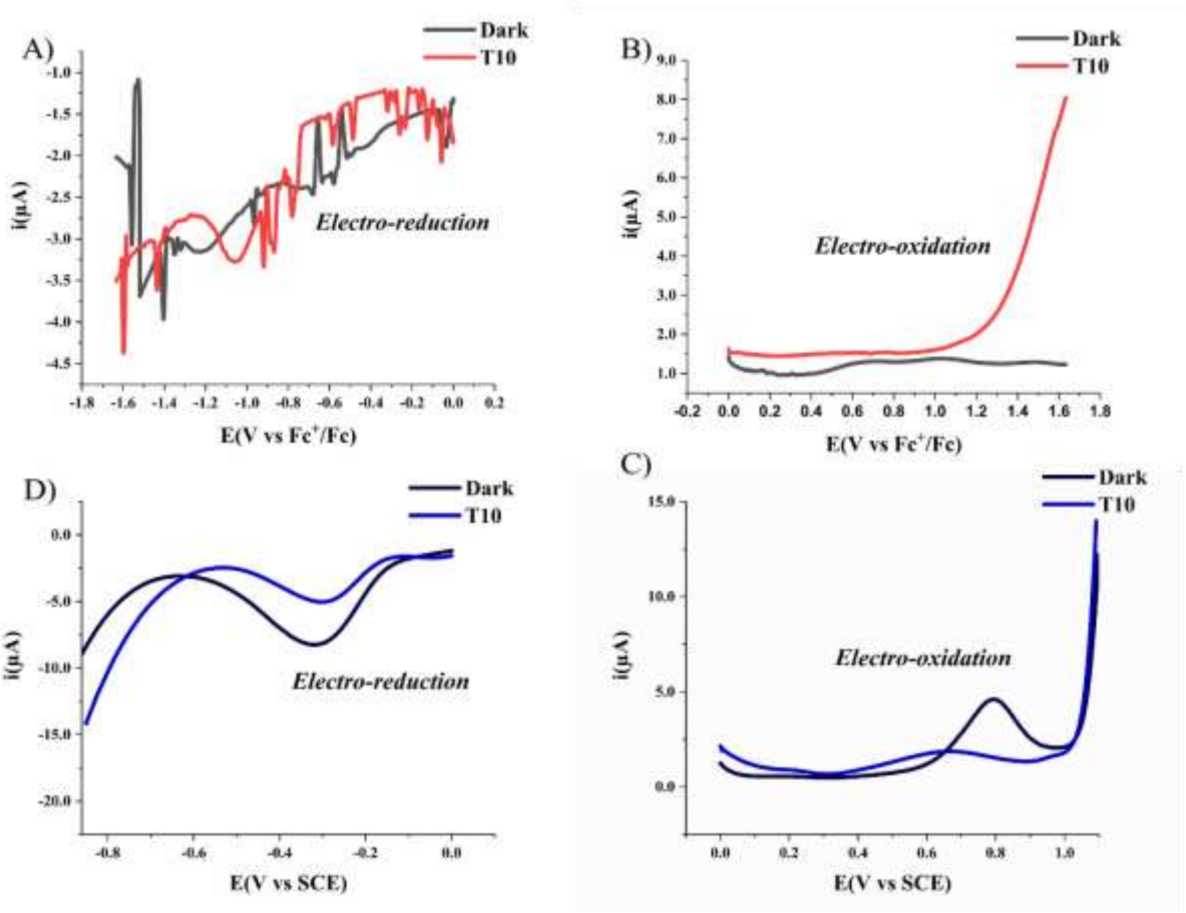

Figure S5. Representative differential pulse voltammetry (DPV) of Gel-1 recorded in dark and after 10 minutes of light irradiation (T10), (A) & (B) redox analysis at Pt electrode in 0.1 mol L^{-1} $(C_4H_9)_4NClO_4$ in DMSO and (C) & (D) redox analysis at Pt electrode in 0.1 M KCl.

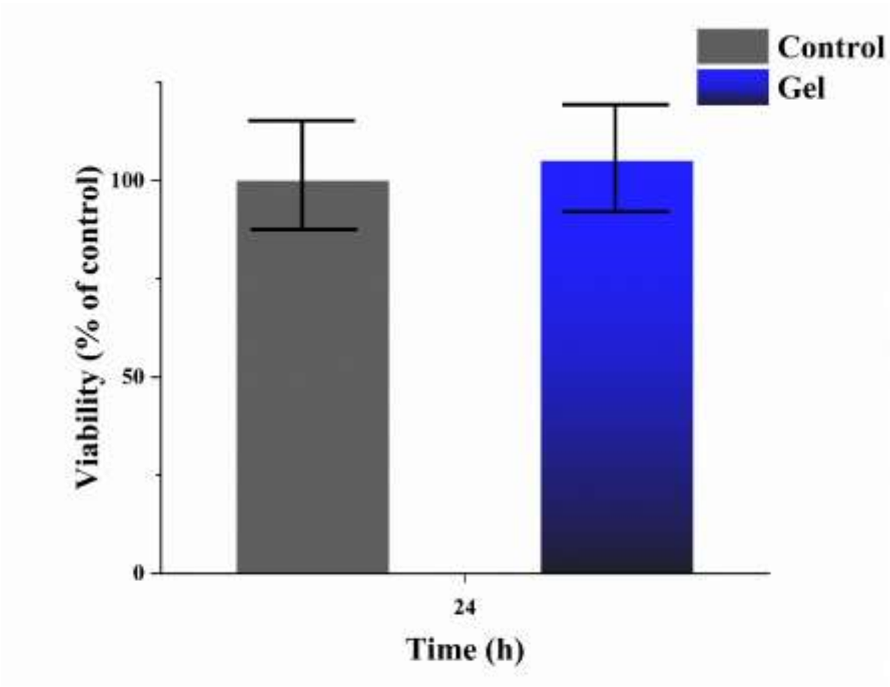

Figure S6. Assessment of cytotoxicity of the Gel-1 against human skin fibroblasts (BJ cell line, ATCC); cell viability after exposure to the gel extract was evaluated by MTT assay, *statistically significant results compared to control by unpaired t-test, $p < 0.05$.
